# Bioinformatics analysis to disclose shared molecular mechanisms between type-2 diabetes and clear-cell renal-cell carcinoma, and therapeutic indications

**DOI:** 10.1038/s41598-024-69302-w

**Published:** 2024-08-19

**Authors:** Reaz Ahmmed, Md. Bayazid Hossen, Alvira Ajadee, Sabkat Mahmud, Md. Ahad Ali, Md. Manir Hossain Mollah, Md. Selim Reza, Mohammad Amirul Islam, Md. Nurul Haque Mollah

**Affiliations:** 1 Bioinformatics Lab (Dry), Department of Statistics, University of Rajshahi, Rajshahi, 6205 Bangladesh; 2 Department of Biochemistry & Molecular Biology, University of Rajshahi, Rajshahi, 6205 Bangladesh; 3 Department of Chemistry, University of Rajshahi, Rajshahi, 6205 Bangladesh; 4 Department of Physical Sciences, Independent University, Bangladesh (IUB), Dhaka, Bangladesh; 5 Division of Biomedical Informatics and Genomics, School of Medicine, Tulane University, 1440 Canal St., RM 1621C, New Orleans, LA 70112 USA; 6 Department of Agricultural and Applied Statistics, Bangladesh Agricultural University, Mymensingh, 2202 Bangladesh

**Keywords:** Clear-cell renal-cell carcinoma, Type-2 diabetes, Shared key genes, Molecular mechanisms, Drug repurposing, Bioinformatics analysis, Cancer, Computational biology and bioinformatics, Drug discovery, Genetics, Molecular biology, Biomarkers, Diseases, Molecular medicine, Oncology

## Abstract

Type 2 diabetes (T2D) and Clear-cell renal cell carcinoma (ccRCC) are both complicated diseases which incidence rates gradually increasing. Population based studies show that severity of ccRCC might be associated with T2D. However, so far, no researcher yet investigated about the molecular mechanisms of their association. This study explored T2D and ccRCC causing shared key genes (sKGs) from multiple transcriptomics profiles to investigate their common pathogenetic processes and associated drug molecules. We identified 259 shared differentially expressed genes (sDEGs) that can separate both T2D and ccRCC patients from control samples. Local correlation analysis based on the expressions of sDEGs indicated significant association between T2D and ccRCC. Then ten sDEGs (CDC42, SCARB1, GOT2, CXCL8, FN1, IL1B, JUN, TLR2, TLR4, and VIM) were selected as the sKGs through the protein–protein interaction (PPI) network analysis. These sKGs were found significantly associated with different CpG sites of DNA methylation that might be the cause of ccRCC. The sKGs-set enrichment analysis with Gene Ontology (GO) terms and KEGG pathways revealed some crucial shared molecular functions, biological process, cellular components and KEGG pathways that might be associated with development of both T2D and ccRCC. The regulatory network analysis of sKGs identified six post-transcriptional regulators (hsa-mir-93-5p, hsa-mir-203a-3p, hsa-mir-204-5p, hsa-mir-335-5p, hsa-mir-26b-5p, and hsa-mir-1-3p) and five transcriptional regulators (YY1, FOXL1, FOXC1, NR2F1 and GATA2) of sKGs. Finally, sKGs-guided top-ranked three repurposable drug molecules (Digoxin, Imatinib, and Dovitinib) were recommended as the common treatment for both T2D and ccRCC by molecular docking and ADME/T analysis. Therefore, the results of this study may be useful for diagnosis and therapies of ccRCC patients who are also suffering from T2D.

## Introduction

**Figure 1:**
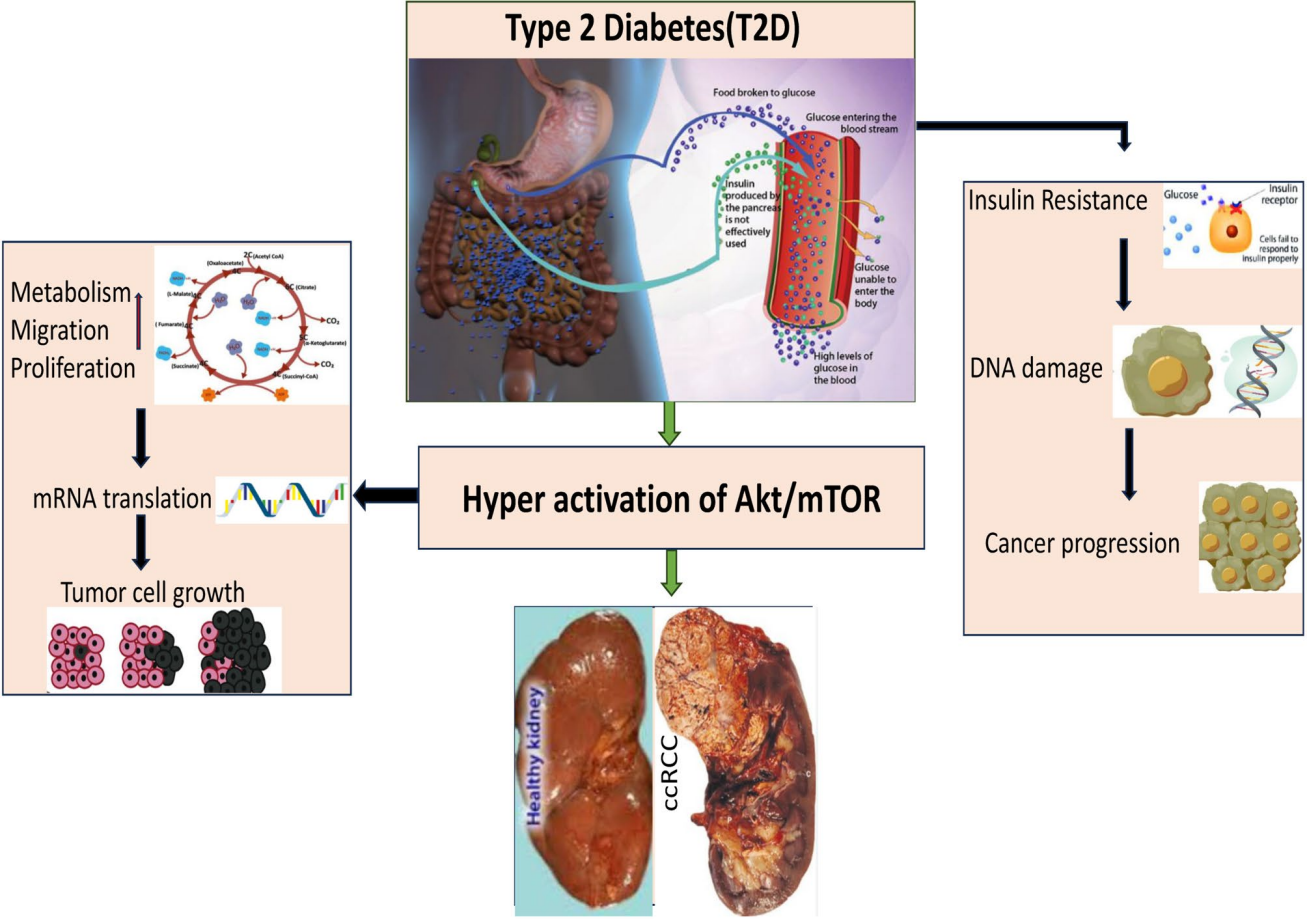
A schematic diagram about the link between T2D and ccRCC.

Cancer is considered as the second leading cause of mortality globally, with around 20 million new cases and more than 10 million deaths annually. Over 50% of cancer patients ultimately died, despite the advancements in the field of diagnosis and therapies^1^. Aged patients are one of the most crucial factors for increasing the cancer-related mortality^2^. The clear-cell renal cell carcinoma (ccRCC) is a common cancer worldwide. There are several types of kidney cancer (KC) including renal cell carcinoma (RCC). The ccRCC is a subtype of RCC, which make up about 70–80% of KC^3^. It is the 8th commonest cancer among women and the 6th most common disease among men^4^. It had the 17th highest cancer-related mortality in 2018 with 175,098 deaths worldwide^5^. In 2020, the death rate of KC patient was around 42%^6^. The ccRCC cancer is the most common type of KC in adults, and its incidence increases with age. While it can occur at any age, the risk of developing ccRCC generally increases after the age of 40, and the highest incidence rates are seen in people aged 60 and older^7^. On the other hand, most of the older peoples suffer from type 2 diabetes (T2D). It is typically occurred due to the insulin resistance^8^. Insulin resistance hinders the body from using glucose for energy, blood sugar levels remain consistently high^9^. A population based study reported that the prevalence of diabetes among all age groups is 2.8% in 2000 and is projected to increase to 4.4% by 2030^10^. However, some other studies have reported that T2D is associated with ccRCC^11–14^, liver cancer^15^, colorectal^16^, breast, stomach, endometrium, pancreas, lymphoid tissue and urinary bladder cancers^11^. A study has been reported that cancer-related deaths account for approximately 13% of overall mortality in diabetic patients^12^. Achieving various kidney problems^13^ including microalbuminuria, macroalbuminuria, or reduced renal function over time affects about 35% to 50% of T2D patients. RCC, is often considered as a metabolic disease, especially ccRCC. It’s target gene mutations associated in metabolic pathways are a clear characteristic of RCC^17^. On the other hand, T2D is also a metabolic disease which is characterized by the deregulation of genes, glucose and lipid metabolism^18^. Insulin resistance also increases the insulin levels, insulin-like growth factors as well as hyperactivation of protein kinase B (Akt)/mTOR in blood which may stimulate the growth and development of tumors^19,20^. Additionally, raised triglyceride levels, higher blood pressure in men, high body mass index (BMI), and T2D in women are distinct risk factors for ccRCC^19^. Numerous genes or proteins that are mutated or methylated for developing cancer, are overexpressed or suppressed, resulting in conformational alterations such as post-translational modifications (PTM). Its results change the cellular signaling pathways and functions which ultimately cause the change of metabolic processes^21^. Thus, ccRCC might be associated with T2D as displayed in Fig. 1 and ccRCC patients may suffer from complicated situations due to the influence of T2D. Therefore, identification of both ccRCC- and T2D-causing shared key genes (sKGs) also known as biomarker genes, is essential in order to investigate their genetic association for better diagnosis and therapies.

During the co-occurrence of T2D and ccRCC, Doctors may be prescribed both diseases specific multiple drugs to the patients^22^. However, drug-drug interaction (DDI) during polypharmacy may create some adverse side effects or toxicity to the patients for which patients may reach to the severe conditions ^23–26^. In that case, Doctors should prescribe fewer numbers of common drugs as the representative of those multiple drugs in order to reduce the toxicity. However, so far, there is no study yet in the literature that has suggested any common drug for the treatment of both diseases though aged patients are at high risk of DDI due to the prevalence of polypharmacy and changes in age-related metabolism. Therefore, it is required to explore potential common drugs for ccRCC and T2D as the representative of those disease specific multiple drugs. In order to explore common drugs, at first, it is required to explore ccRCC- and T2D-causing sKGs as the targets of common drugs, since specific disease-causing key genes/proteins are widely used as the targets of disease specific drugs ^27–30^. Nevertheless, it is very difficult to explore ccRCC- and T2D-causing top-ranked sKGs and candidate therapeutic ligands/agents from huge number of alternatives through the wet-lab experiments only, since wet-lab experiments are time consuming, laborious and costly. To overcome this issues, *in-silico* bioinformatics and system biology approaches are playing the significant roles^31,32^. In the case of target selection, genomics/transcriptomics analysis through integrated statistics and network-based approaches are widely used^31,32^. There are some *in-silico* studies that explored T2D- and ccRCC-causing key genes (KGs) and their pathogenetic mechanisms individually^33–38^. Though, some studies investigated shared KGs (sKGs) for T2D with HCC(Hepatocellular-carcinoma)^39,40^ and CRC (colorectal cancer)^41,42^, however, so far, there is no study in the literature that has explored T2D- and ccRCC-causing sKGs. Therefore, this study aimed to explore both T2D- and ccRCC-causing sKGs highlighting their pathogenetic mechanisms and candidate common drug molecules for taking a better treatment plan against ccRCC with T2D, by using the integrated bioinformatics and system biology approaches.

## Materials and methods

### Data source and descriptions

To explore shared key genes (sKGs) between T2D stimulates ccRCC, we considered four micro-array gene expression profile datasets for each of T2D (GSE25724^43^, GSE29221^44^, GSE29226^45^ and GSE29231^46^) and ccRCC (GSE66270^47^, GSE66272^48^, GSE76351^49^ and GSE66271^50^) from the Gene Expression Omnibus (GEO) platform in the National Center for Biotechnology Information (NCBI) database. Table 1 provides the detailed descriptions of the datasets.

**Table 1:** Data source and descriptions.

GEO datasets	Country	Platform	Cases	Control
GSE25724	Italy	GPL96[HG-U133A] Affymetrix Human Genome U133A Array	6(T2D)	7
GSE29221	India	GPL6947Illumina HumanHT-12 V3.0 expression beadchip	12(T2D)	12
GSE29226	India	GPL6947Illumina HumanHT-12 V3.0 expression beadchip	12(T2D)	12
GSE29231	India	GPL6947Illumina HumanHT-12 V3.0 expression beadchip	12 (T2D)	12
GSE66270	Germany	GPL570[HG-U133_Plus_2] Affymetrix Human Genome U133 Plus 2.0 Array	14(ccRCC)	14
GSE66272	Germany	GPL570[HG-U133_Plus_2] Affymetrix Human Genome U133 Plus 2.0 Array	27(ccRCC)	27
GSE76351	Russia	GPL11532[HuGene-1_1-st] Affymetrix Human Gene 1.1 ST Array [transcript (gene) version]	12(ccRCC)	12
GSE66271	Germany	GPL570 [HG-U133_Plus_2] Affymetrix Human Genome U133 Plus 2.0 Array	13(ccRCC)	13

### Identification of Differentially Expressed Genes (DEGs)

To identify differentially expressed genes (DEGs) between case and control groups, we considered the, since it shows good performance in the case of small sample sizes also. It produces *P*.values based on the moderated *t*-statistic^51^ to measure the significance of differential expressions between two condition. The moderated t-statistic is formulated by combining the classical and Bayesian estimation of the relevant parameters^51,52^. Then *g*th differentially expressed gene (DEG_*g*_) is defined by combining its adjusted *P*.value and the average of log2 fold-change (aLog_2_FC) values as follows,

$${\text{\;DE}}{{\text{G}}_g} = \left\{ {\begin{array}{*{20}{l}} {{\text{DEG}}\left( {{\text{Up}}} \right),\;}&{{\text{if\;adj}}.P.{\text{value}}\left\langle {0.05\;{\text{and}}\;aLo{g_2}\left( {{\text{F}}{{\text{C}}_g}} \right)} \right\rangle + 1} \\ {{\text{DEG}}\left( {{\text{Down}}} \right),}&{{\text{if\;adj}}.P.{\text{value}} < 0.05\;{\text{and}}\;aLo{g_2}\left( {{\text{F}}{{\text{C}}_g}} \right) < - 1} \end{array}} \right.$$

where alog_2_FC value for *g*th gene is computed as

1 $$aLo{g_2}F{C_g} = \left\{ {\begin{array}{*{20}{l}} {\frac{1}{{n_1}}\mathop \sum \limits_i^{n_1} lo{g_2}(z_{gi}^D) - \frac{1}{{n_2}}\mathop \sum \limits_j^{n_2} lo{g_2}\left( {z_{gj}^C} \right),}&{if\,{n_1} \ne {n_2}} \\ {\frac{1}{n}\mathop \sum \limits_i^n lo{g_2}\left( {\frac{{z_{gi}^D}}{{z_{gj}^C}}} \right),}&{if\,{n_1} = {n_2}\; = n\;} \end{array}} \right.$$

Here $z_{gi}^D$ and $z_{gj}^C$ are the responses/expressions for the *g*th gene with the *i*th disease and *j*th control samples, respectively. We utilized the limma R-package^53^ for calculating the *P*.values and Log_2_FC values to select the DEGs, significantly for both T2D and ccRCC patients.

### Identification of shared DEGs (sDEGs)

At first, we detected DEGs between ccRCC and control samples based on four datasets with NCBI accession ID GSE66270, GSE66272, GSE76351, and GSE66271. Then detected DEGs for T2D vs. control samples were detected based on four datasets with accession ID GSE25724, GSE29221, GSE29226 and GSE29231. Then shared DEGs (sDEGs) that are able to separate both T2D and ccRCC samples from the control samples, were selected.

#### Local genetic association between T2D and ccRCC through sDEGs

Though average of log_2_FC (alog_2_FC) values were calculated for T2D and ccRCC from independent datasets by Eq. 1, but these values were calculated from the same unit of sDEGs for each of T2D and ccRCC. A shared DEG (sDEG) is called upregulated for two or more diseases if alog_2_FC > 0 and downregulated if alog_2_FC < 0. If we assume that the function of a gene is almost same for all control patients, we may measure the genetic association between any two diseases X and Y based on their alog_2_FC values corresponding to the expressions of sDEGs through the Pearson’s correlation coefficient which is defined as

2 $${r_{xy}} = \frac{{\sum \left( {{x_g} - \bar x} \right)\left( {{y_g} - \bar y} \right)}}{{\sqrt {\sum {{({x_g} - \bar x)}^2}{{\left( {{y_g} - \bar y} \right)}^2}} }}$$

where ${x_g} = alo{g_2}FC\left( X \right)$ and ${y_g} = alo{g_2}FC\left( Y \right)$ are the alog_2_FC values of the *g*^th^ gene for the two diseases *X* and *Y*; $\bar x\;$ and $\bar y\;$ are the means of ${x_g}^{\prime}s$ and ${y_g}^{\prime}s$, respectively.

### Identification of shared key genes (sKGs) from sDEGs

Proteins interact with other proteins in the cell to carry out their tasks, and information generated by the protein–protein interaction (PPI) network is used to select the key genes^54,55^. In order to generate PPI network, the distance matrix ‘D’ is calculated as

$${\text{D}}\left( {{\text{i}},{\text{j}}} \right) = \frac{{2\left| {{N_i} \cap {N_j}} \right|}}{{|{N_i}\left| { + |{N_j}} \right|}},$$

where *N*_*i*_ is the neighbor set of *i*th protein and *N*_*j*_ is the neighbor set of *j*th protein. In order to identify shared key genes (sKGs), a PPI-network of sDEGs was constructed using the STRING database^56^. To select the sKGs from the PPI network, we used different topological measures (Betweenness^57^, Degree^58^, BottleNeck^59^, Closeness^60^ MNC^61^, Radiality^62^ and Stress^63^) by using CytoHubba plugin-in Cytoscape software^64^.

### *In-silico* validation of sKGs using independent datasets and databases

The differential expression patterns of sKGs were validated in both disorders (ccRCC & T2D) by Box plots analysis with the independent datasets from NCBI, TCGA and GTEx databases. We used the TCGA and GTEx databases in the GEPIA2^65^ web-tool to confirm the differential expression patterns of sKGs between ccRCC and control samples. In ordered to validate the differential expression patterns of sKGs between T2D and control samples, we used two independent datasets with accession IDs GSE15932^66^ and GSE20966^67^ from NCBI database.

### Regulatory network analysis of sKGs

A gene regulatory network (GRN) displays molecular regulators that interact with each other in the cell to control the gene expressions. The transcription factors (TFs) and microRNAs (miRNAs) are considered as the transcriptional and post-transcriptional regulators of protein coding genes. To select the top-ordered TFs as the key transcriptional regulators of sKGs, the TFs versus sKGs interaction network analysis was performed by using JASPAR^68^ databases with the NetworkAnalyst web-tool^69^. Similarly, to identify top-ordered miRNAs as the key post-transcriptional regulators of sKGs, the sKGs versus miRNAs interaction network analysis was performed by using the TarBase database^70^ databases with the NetworkAnalyst web-tool^69^.

### The sKGs-set enrichment analysis with GO-terms and KEGG-pathways

The sKGs-set enrichment studies with gene ontology (GO) terms and Kyoto encyclopedia of genes and genomes (KEGG) pathways^71^ were performed to explore biological processes (BP), molecular functions (MF), cellular components (CC) and pathways of sKGs. In order to identify significantly enriched GO terms (BPs, MF, CCs) or KEGG-pathways by the sKGs-set, a 2 × 2 contingency table was constructed (see Table 2).

**Table 2:** A 2 × 2 Contingency table.

Annotated genes	sKGs (proposed)	Not-sKGs	Marginal total
Annotated gene-set in *i*^th^ GO term/KEGG pathway (*A*_*i*_)	*k*_*i*_	*M*_*i*_*—k*_*i*_	*M*_*i*_
Complement gene-set of *A*_*i*_* (*${A}_{{\varvec{i}}}^{c}$)	*n—k*_*i*_	*N—M*_*i*_* – n* + *k*_*i*_	*N—M*_*i*_
Marginal total	*n*	*N—n*	*N* (Grand total)

where *A*_*i*_: annotated genes in the *i*^th^ BPs/MFs/CCs/KEGG-pathways in the database, *M*_*i*_: total number of annotated genes in *A*_*i*_ (*i* = 1, 2,…,*r*); *N*: total number of annotated genes in $A = \mathop{\cup }\limits_{i = 1}^r {A_{\text{i}}} = {A_i} \cup A_i^c$ such that $N \leqslant \mathop \sum \limits_{i = 1}^r {M_i}.$ Here *n*: total number of sKGs, *k*_*i*_: number of sKGs belonging to *A*_*i*_. To detect the significantly enriched GO-terms or KEGG-pathways with sKGs, the database for annotation, visualization and integrated discovery (DAVID)^72^ was used to calculate the *p*-value by the Fisher exact test statistic based on hypergeometric distribution^73^.

### DNA methylation analysis

Development of many diseases including cancers, obesity and T2D are associated the aberrant DNA methylation. DNA methylation analysis is used to gain relevant knowledge about gene regulation and detect potential biomarkers. In this study, MethSurv^74^ and UALCAN^75^ were employed to investigate the DNA methylation status of sKGs. DNA methylation level was expressed as β-values (which ranged from 0 to 1). Using the equation M / (M + U + 100), the -values are determined. Here, M and U are stand for fully methylated and totally unmethylated intensities, respectively.

### Exploring sKGs-guided repurposable common drug molecules for both T2D and ccRCC

There are two *in-silico* ways (de-novo and repurposing) of exploring drug molecules for diseases, where de-novo approach is time consuming, costly and laborious compared to the drug repurposing (DR) approach, since the DR approach explores existing drugs for a disease of interest that drugs are already approved for other diseases^76^. However, in both-approaches, molecular docking analysis with the synthetic molecules ^30,77^ as well as phytocompounds^78–80^ are widely used in order to explore potential ligands/agents. In order to explore sKGs-guided repurposable common drug molecules for T2D and ccRCC, we collected 148 candidate molecules from published articles associated with T2D and ccRCC, and online databases as given in Table S1. The Protein Data Bank(PDB)^81^, SWISS-MODEL^82^ and AlphaFold databases were utilized to obtain the three-dimensional configurations of every sKGs-mediated receptor proteins. Using Swiss PDB view^83^ and AutoDock Vina^84^, receptor-proteins were pre-processed by including charges and reducing energy, respectively. All 148 potential drug compounds' 3D structures were downloaded from the PubChem database^85^ and ready for molecular docking simulation by using AutoDock tools 1.5.7 to set the ligand's rotatable/non-rotatable links and torsion tree. Then AutoDock Vina^84^ was used to compute the binding affinities between the drugs and the target proteins. The docked complexes were examined using PLIP^86^, PyMol^87^, and Discovery Studio Visualizer (BIOVIA 2021) software^88^ to determine the types, distances, and surface complexes of non-covalent bonds. Let B_*ij*_ indicates the BAS (binding affinity score) between *i*^th^ receptors (*i* = 1, 2, …, p) and j^th^ ligands/agents (*j* = 1, 2, …, q). Then receptors were arranged according to the decreasing-order of row average $\left( {\frac{1}{p}\mathop \sum \limits_{{\text{j}} = 1}^{\text{q}} {{\text{B}}_{ij}},\;i = 1,2 \ldots p} \right)$ and ligands/agents according to the decreasing-order of column average $\left( {\frac{1}{q}\mathop \sum \limits_{{\text{i}} = 1}^{\text{p}} {{\text{B}}_{{\text{ij}}}},\;j = 1,2, \ldots ,q{\text{\;}}} \right)$ to select the top-ranked few agents/ligands as the potential candidate drug molecules.

### *In-silico* validation of candidate drug molecules by ADME/T analysis

The drug-like characteristics and ADMET (absorption, distribution, metabolism, excretion, and toxicity) properties were determined of top-ranked 3 drug compounds in order to learn more about their structural characteristics and chemical descriptors. We use SCFBio (http://www.scfbio-iitd.res.in/software/drugdesign/lipinski.jsp) web tool for evaluating their Lipinski rule satisfaction of drug likeness properties (including molecular weight, number of hydrogen donor and acceptor bonds, rotatable bond, octanol/water partition coefficient or LogP value, etc.)^89^. The ADMET properties were then predicted by using the online databases SwissADME^90^ and, and pkCSM^91^. The ADME/T calculations of medicinal compounds were performed using their optimal structures in SMILES formats.

## Results

### Identification of Differentially Expressed Genes (DEGs)

At first, we identified DEGs for both T2D and ccRCC patients by using LIMMA with an r-package. The cut-off at adjusted *P*.values > 0.05 and |Log_2_FC|> 1 was used to select the DEGs as mentioned in section "Identification of Differentially Expressed Genes (DEGs)"**.** For ccRCC, we detected 15,348, 14,472, 1820 and 1576 downregulated DEGs, and 8150, 8166, 563 and 759 upregulated DEGs, for the NCBI datasets with accession ID GSE66270, GSE66272, GSE76351, and GSE66271, respectively. Then, 738 upregulated and 47 downregulated DEGs (Table S2) were detected as common DEGs (cDEGs) for ccRCC. From the NCBI datasets with accession ID GSE25724, GSE29221, GSE29226, and GSE29231, we identified 2651, 459, 839, and 2854 upregulated DEGs, and 3032, 1875, 2173 and 1569 downregulated DEGs respectively, for T2D patients. We found 252 downregulated and 498 upregulated cDEGs for T2D (Table S3).

### Identification of shared DEGs (sDEGs) between T2D and ccRCC

In the previous section, we found 738 upregulated and 47 downregulated DEGs for ccRCC based on four transcriptomics datasets. Similarly, 252 downregulated and 498 upregulated DEGs for T2D based on another four transcriptomics datasets. Then we detected 194 as upregulated shared DEGs (sDEGs) and 65 as downregulated sDEGs for both T2D and ccRCC (Table S4 & S5). Thus, we considered in total 259 sDEGs for both T2D and ccRCC.

### Local association between T2D and ccRCC through sDEGs

To find the link between T2D and ccRCC, we computed local correlation coefficient between T2D and ccRCC based on the aLog_2_FC values of sDEGs by using Eq. 2. The correlation coefficient was found with a value of 0.82, which indicates that T2D and ccRCC are locally associated with each other through the expressions of sDEGs.

### Identification of shared Key Genes (sKGs)

**Figure 2:**
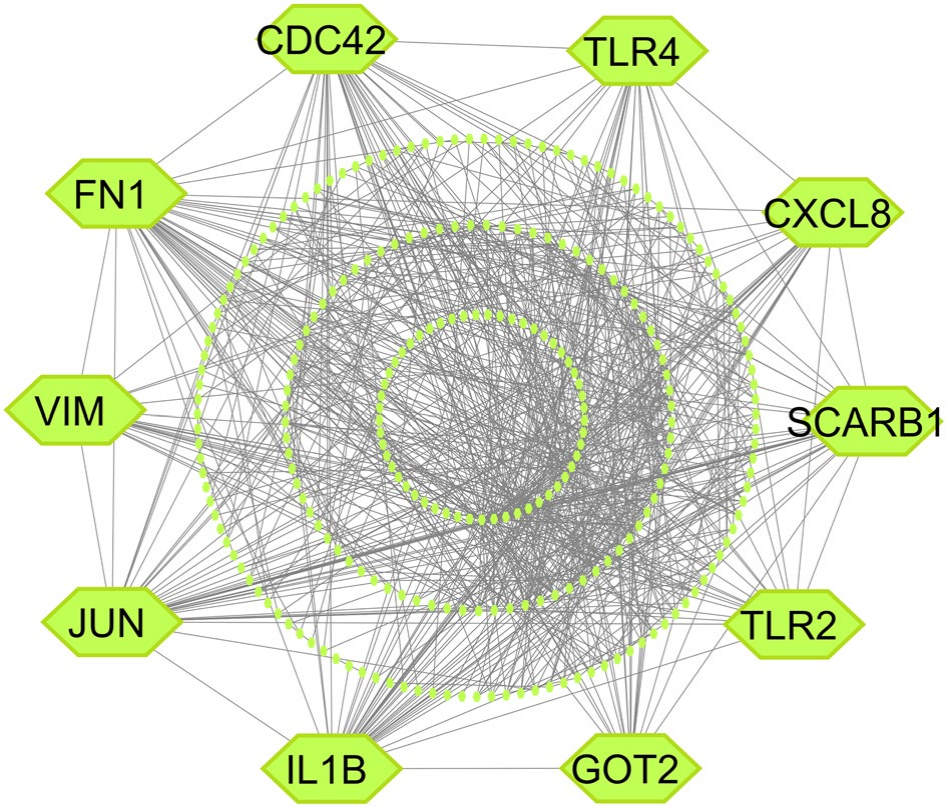
Protein–protein interaction (PPI) network of sDEGs to identify sKGs, where the chartreuse color nodes indicated the sKGs.

The STRING database was used to build the PPI network of sDEGs which has 259 nodes and 773 edges (Fig. 2) By using seven topological measures (Betweenness, BottleNeck, Closeness, Degree, MNC, Radiality and Stress) in the PPI network, we chose the top 10 cHubGs (VIM, CDC42, SCARB1, CXCL8, FN1, IL1B, JUN, TLR2, TLR4 and GOT2) (Table S6).

### *In-silico* validation of sKGs using independent datasets and databases

We investigated the differential expression patterns of sKGs between ccRCC and control samples through Box-plot analysis based on the independent gene expression profiles from TCGA and GTEx databases that contained 523 ccRCC and 100 control samples. From Figure S1A, we observed that 3 sKGs (CDC42, GOT2, CXCL8) are downregulated and the remaining 7 sKGs (TLR4, IL1B, TLR2, FN1, JUN, VIM, SCARB1) are upregulated, which supported the proposed results. We also investigated the differential expression patterns of sKGs between T2D and control samples through Box-plot analysis based on the independent gene expression profiles from the NCBI database with accession ID GSE15932 and GSE20966, where the dataset with accession ID GSE15932 contains 8 pancreatic cancer, 8 T2D and 8 control samples. In our analysis, we considered only T2D and control samples. Figure S1B shows that 3 sKGs (CDC42, GOT2, and CXCL8) are downregulated in T2D, while the rest 7 sKGs (TLR4, IL1B, TLR2, FN1, JUN, VIM, and SCARB1) are upregulated, which also supported the proposed results.

### The regulatory network analysis of sKGs

**Figure 3:**
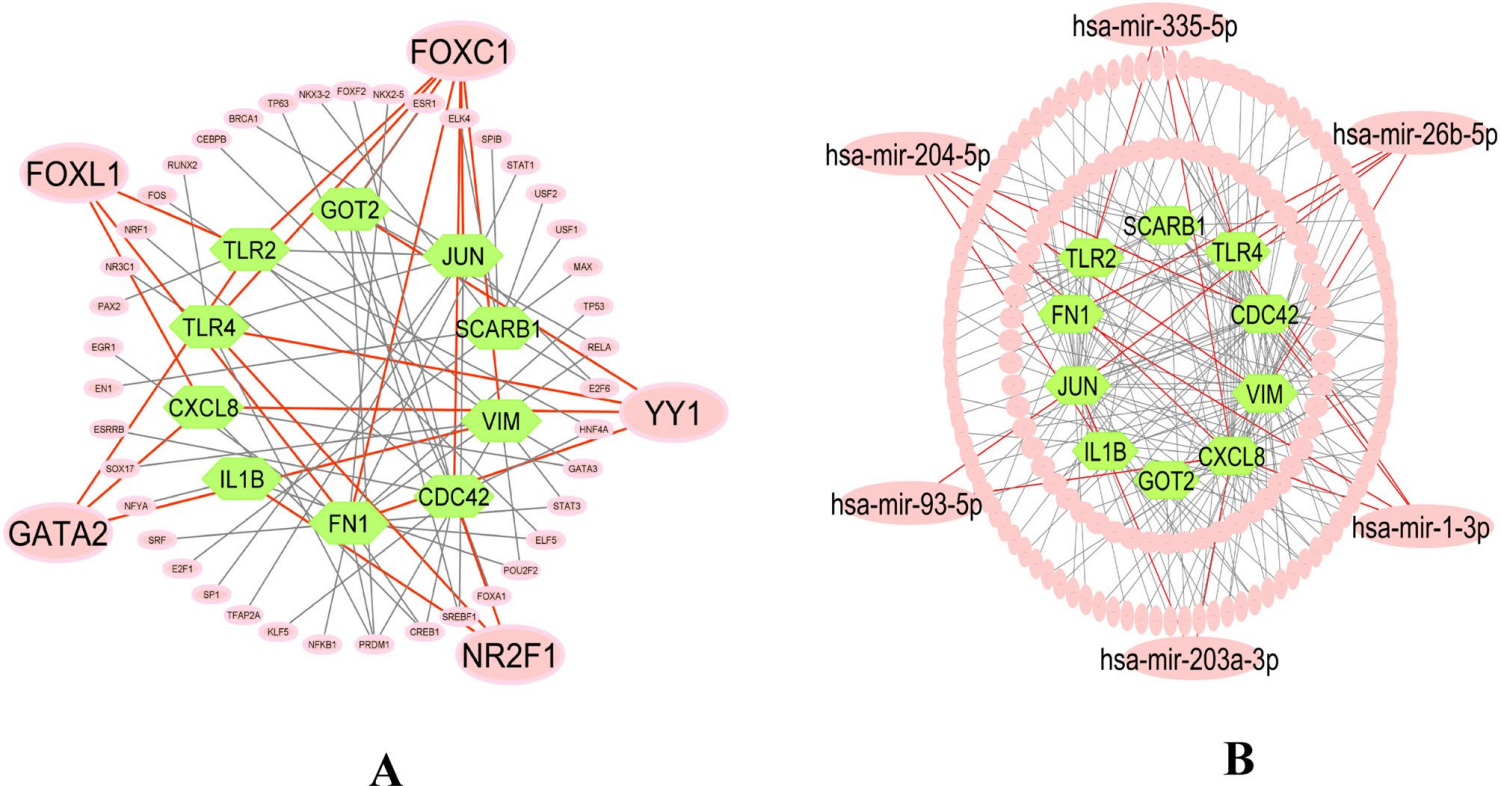
(**A**) The sKGs-TFs interaction network based on JASPAR database (**B**) The miRNA-sKGs interaction network based on TarBase database.

The top-ranked five significant TFs proteins (FOXL1, FOXC1, NR2F1, YY1 and GATA2) and micro-RNAs (hsa-mir-93-5p, hsa-mir-203a-3p, hsa-mir-204-5p, hsa-mir-335-5p, hsa-mir-26b-5p, and hsa-mir-1-3p) were identified as the key transcriptional and post-transcriptional regulators of sKGs by using the TFs-sKGs-miRNAs interaction network analysis (see Fig. 3).

### sDEGs-set enrichment analysis with GO-terms and KEGG pathway

We carried out GO and KEGG pathway enrichment analysis for 10 sKGs to look into the shared pathogenetic mechanisms between T2D and ccRCC. The top five MFs, BPs, CCs, and KEGG pathways are listed in Table 3. Significantly enhanced KEGG pathways and GO terms with sDEGs through the involvement of sKGs linked to the pathogenetic mechanisms of T2D on ccRCC.

**Table 3:** Significantly enriched GO-terms and KEGG pathways that are associated with T2D and ccRCC.

	GO ID	GO-Terms	sDEGs (counts)	*P.v*alue	Associated sKGs
Biological process (BPs)	GO:0,006,915	apoptotic process	22	3.07E−04	IL1B, TLR2
	GO:0,007,165	signal transduction	28	1.95E−06	CXCL8, IL1B, TLR2
	GO:0,006,954	inflammatory response	45	1.25E−21	CXCL8, TLR2, TLR4
	GO:0,010,628	positive regulation of gene expression	19	7.46E−04	FOXL1, FOXC1, CXCL8, FN1, IL1B, TLR2, TLR4
	GO:0,034,976	Response to endoplasmic reticulum stress	8	6.08E−04	JUN, CXCL8
Molecular Function (MFs)	GO:0,005,178	integrin binding	15	2.26E−06	FN1, IL1B
	GO:0,042,802	identical protein binding	44	0.001422588	JUN, FN1, VIM, CDC42, TLR2, TLR4
	GO:0,008,201	heparin binding	10	0.001969	CXCL8, FN1
	GO:0,001,875	lipopolysaccharide receptor activity	3	0.003454	SCARB1, TLR4, TLR2
	GO:0,005,515	Protein binding	248		JUN, FN1, TLR2, TLR4, IL1B, SCARB1, CXCL8, CDC42, VIM,
Cellular Components (CC)	GO:0,070,062	extracellular exosome	64	3.45E−07	SCARB1, VIM, FN1, GOT2
	GO:0,005,829	cytosol	114	5.04E−05	CDC42, IL1B, VIM, NR2F1
	GO:0,009,986	cell surface	19	0.007489	SCARB1, TLR2, TLR4,
	GO:0,016,020	membrane	113	1.31E−04	TLR2, TLR4, FN1
	GO:0,005,604	Basement membrane	9	5.59E−05	FN1

	Hsa ID	KEGG terms	sDEGs (counts)	*P.v*alue	Associated sKGs
KEGG Pathway	hsa05169	Epstein-Barr virus	14	1.65E−04	TLR2, VIM, JUN
	hsa004621	NOD-like receptor signaling pathway	13	2.87E−04	JUN, CXCL8, IL1B, TLR4
	hsa05323	Rheumatoid arthritis	9	4.61E−04	JUN, CXCL8, IL1B, TLR2, TLR4
	hsa05417	Lipid and atherosclerosis	13	0.00105	JUN, CXCL8, CDC42, IL1B, TLR2
	hsa05165	Human papillomavirus infection	18	2.60E−04	FN1, CDC42

### Disease enrichment analysis with sKGs

**Figure 4:**
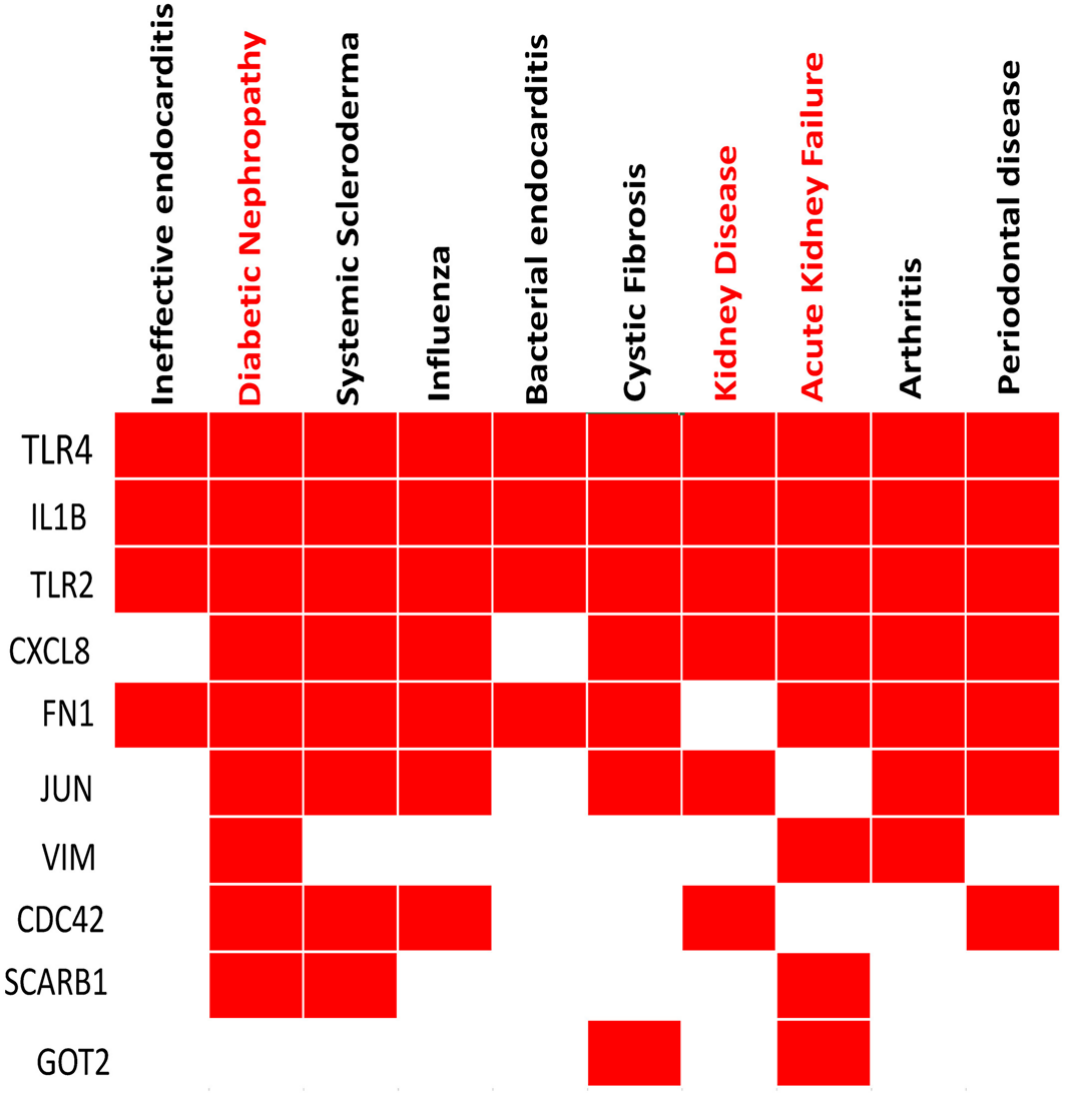
Results of disease enrichment analysis with sKGs, where red box indicates significant association (*p*-value < 0.05).

We performed disease enrichment analysis with sKGs by using the Enrichr web-tool with DisGeNET database to investigate the association of sKGs with different diseases. This analysis significantly detected top-ranked 10 diseases including Diabetic Nephropathy, Kidney Failure and Kidney Disease (see Fig. 4) that are associated with sKGs.

### DNA methylation analysis of sKGs in ccRCC

DNA methylation is an epigenetic mechanism which regulates gene expression by recruiting proteins involved in gene repression or by inhibiting the binding of transcription factor(s) to DNA^75^. Significant tumor suppressor gene silencing is facilitated by DNA hypermethylation, which primarily happens at the CpG islands within a gene's promoter region. On the other hand, oncogenes are upregulated when DNA hypomethylation occurs ^92^.Therefore, we examined DNA methylation status at CpG sites for the sKGs (CDC42, SCARB1, GOT2, CXCL8, FN1, IL1B, JUN, TLR2, TLR4, and VIM) by methsurv. We observed that ten sKGs had significant CpG sites (p-value of ≤ 0.001) Table-S7. Additionally, UALCAN was also utilized to visualize promoter methylation status of the 10 sKGs in ccRCC. From Box whisker plot it was found that seven sKGs (SCARB1, FN1, IL1B, JUN, TLR2, TLR4, and VIM)) were hypomethylated according to β-values (ranging from 0 (that means completely unmethylated) to 1 (that means highly methylated)) which is strong evidence that, seven sKGs were upregulated in ccRCC**.**

### Drug repurposing by molecular docking

**Figure 5:**
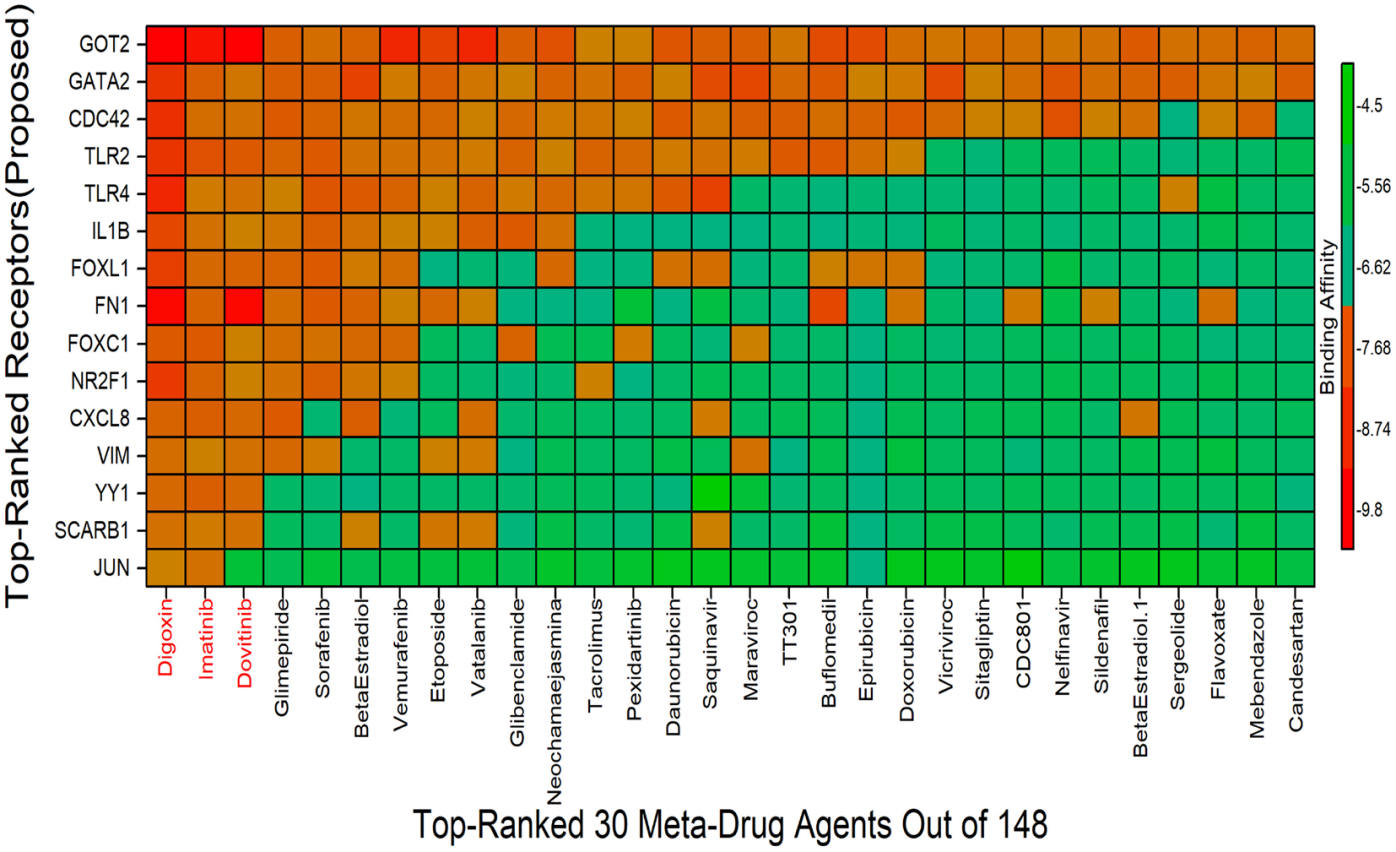
Molecular docking scores, where red color indicates strong drug-target binding. Image of score matrix, where X-axis indicates top-ordered 30 drug agents (out of 148) and Y-axis indicates ordered proposed receptors.

To explore candidate ligands (drug molecules) for the treatment against T2D and ccRCC, we considered our proposed 10 sKGs and their regulatory 5 TFs proteins as the receptors. We collected the data from two distinct sources in order to obtain the 3D structures of these receptors. The Protein Data Bank (PDB) was searched for the structures of 10 receptors (CDC42, SCARB1, GOT2, CXCL8, FN1, IL1B, JUN, TLR2, TLR4, and VIM) using the following PDB codes:1a4r, 5ktf, 5ax8, 3il8, 1e88, 2KH2, 1jun, 2z80, 2z62 and 1gk7. The "AlphaFold Protein Structure Database" was used to collect the remaining five targets (FOXL1, FOXC1, NR2F1, YY1, and GATA2). We computed the binding affinity scores (BAS), between the proposed receptors and the candidate drug molecules (Table S1) by using molecular docking analysis. To select the top-ranked therapeutic candidates, drug molecules were ordered based on the average BAS across the receptors (Table S8). Similarly, receptors were ordered based on the average BAS across the drug molecules. Figure 5 displayed the top-order 30 drug molecules corresponding to the ordered receptors. We observed that 3 molecules Digoxin, Imatinib and Dovitinib produces average BAS < -7.7 kcal/mol, but the other molecules satisfy BAS > -7.7 kcal/mol. Therefore, we considered Digoxin, Imatinib and Dovitinib as the top-ranked drug molecules to inhibit the proposed sKGs. It is seen that top two molecules Digoxin and Imatinib strongly binds (BAS < -7.0 kcal/mol) to all of the receptor proteins. The third top-ranked molecule Dovitinib also strongly binds to all of the receptor proteins except JUN. Therefore, the proposed three drug molecules might be the potential inhibitors against the T2D- and ccRCC-causing genes.

### *In-silico* drug validation

The ADME properties of a drug molecule were used to evaluate its absorption, distribution, metabolism, excretion, and toxicity. The drug likeness properties of a drug molecules explore its physicochemical descriptor to describe its different kind of chemical properties (Table 4). According to the Lipinski rule we found that, two drugs (Imatinib and Dovitinib) follow all rule-of-five (ROF), the rest one (Digoxin) violates three rules (MW, HBA, and HBD) from ROF. The lipophilicity (LogP value) of these 3 drugs supports the standard range (1—less than equal 5)^89^ of Lipinski’s rule. These are found as lipophilic compounds based on their LogP values compared with the standard values. Thus, our suggested top-ranked 3 drug molecules have fulfilled almost all the drug-likeness criteria (Table 4A). The ADME and toxicity analysis of proposed compounds can be examined through various parameter for evaluating its effectiveness and indemnification. The compounds are predicted to have sufficient absorption in the gastrointestinal tract, making them a promising oral drug candidate. A compound with a high Human Intestinal Absorption (HIA) score HIA ≥ 30%, is considered to be highly absorbed in the human intestine^93,94^. In our proposed 3 drugs we found that, all the 3 compounds have high HIA score ≥ 50% which indicate that they have good absorption properties by the human body and also our top-ranked 2 drugs have the ability to inhibit the P-glycoprotein inhibitor (P-gpI) except the Dovitinib. The ability of a compound to cross the blood–brain barrier (BBB) is determined by BBB-permeability index. Compounds having a LogBB ≥ 0.3 can cross the BBB easily and potentially while the value with LogBB < -1 are considered to be poorly distributed through to the BB barrier. All the three compounds evaluated and found that all proposed drugs poorly able to cross the BBB (TTable 4B). As well as, according to the value of LogPS (CNS) are considered to partly penetrate the central nervous system. Membrane-bound hemoproteins called human cytochrome P 450 (CYP) enzymes play an essential role for homeostasis, drug detoxification, and cellular metabolism. About 50% of all common clinical medication elimination in humans and almost 80% of the oxidative metabolism are attributed to more than one CYPs from CYP classes 1–3^95^. All the 3 drugs except Digoxin have the YES properties to inhibit the CYP3A4 membrane of our human body. The highest value of oral toxicity or lethal dose (LD_50_) were found as 3.7 mol/kg for digoxin, whereas 2.9 and 2.4 mol/kg was identified as lowest value for imatinib and dovitinib respectively. The greater the value of LC_50_ the lower the toxicity of a drug molecules, while a smaller value indicates higher toxicity. Thus, from the given table we can observed that the order of the LC_50_ value for the top-ranked drugs are digoxin, imatinib, and dovitinib respectively. Toxicity analyses (AMES, Minnow toxicity (LC_50_), and lethal dose LD_50_) of our suggested revealed that these compounds were inactive for all of the toxicity prediction parameters utilized, and consequently were predicted to be non-toxic.

**Table 4:** Drug likeness and ADME/T analysis results. (A) Drug likeness profile of candidate drug molecules. (B) ADME and Toxicity (ADME/T) profile of top-ranked 3 drug molecules.

A. Drug likeness profile of candidate drug molecules										
Compounds	Molecular weight	Log P		H-bond Acceptor (HBA)	H-bond donor (HBD)		Polar Surface area (Å^2^)		No of rotatable bond	
Digoxin	780	4.84		14	6		203.06		7	
Imatinib	493	4.04		6	2		86.28		8	
Dovitinib	393.43	2.26		4	3		94.04		2	

B. ADME and Toxicity (ADME/T) profile of top-ranked 3 drug molecules										
Compounds	Absorption			Distribution		Metabolism	Excretion	Toxicity		
	Caco2 Permeability	HIA (%)	P-gpI	BBB (LogBB)	CNS LogPS	CYP3A4 Inhibitor	TC	AMES	LC_50_ (log mM)	LD_50_ (mole/kg)
				(Permeability)						
Digoxin	0.59	68.58	Yes	− 1.39	− 3.81	No	3.67	No	4.35	3.7
Imatinib	1.09	93.85	Yes	− 1.37	− 2.51	Yes	0.72	No	2.08	2.9
Dovitinib	0.47	83.63	No	− 0.71	− 2.27	Yes	0.76	Yes	3.04	2.4

## Discussion

**Figure 6:**
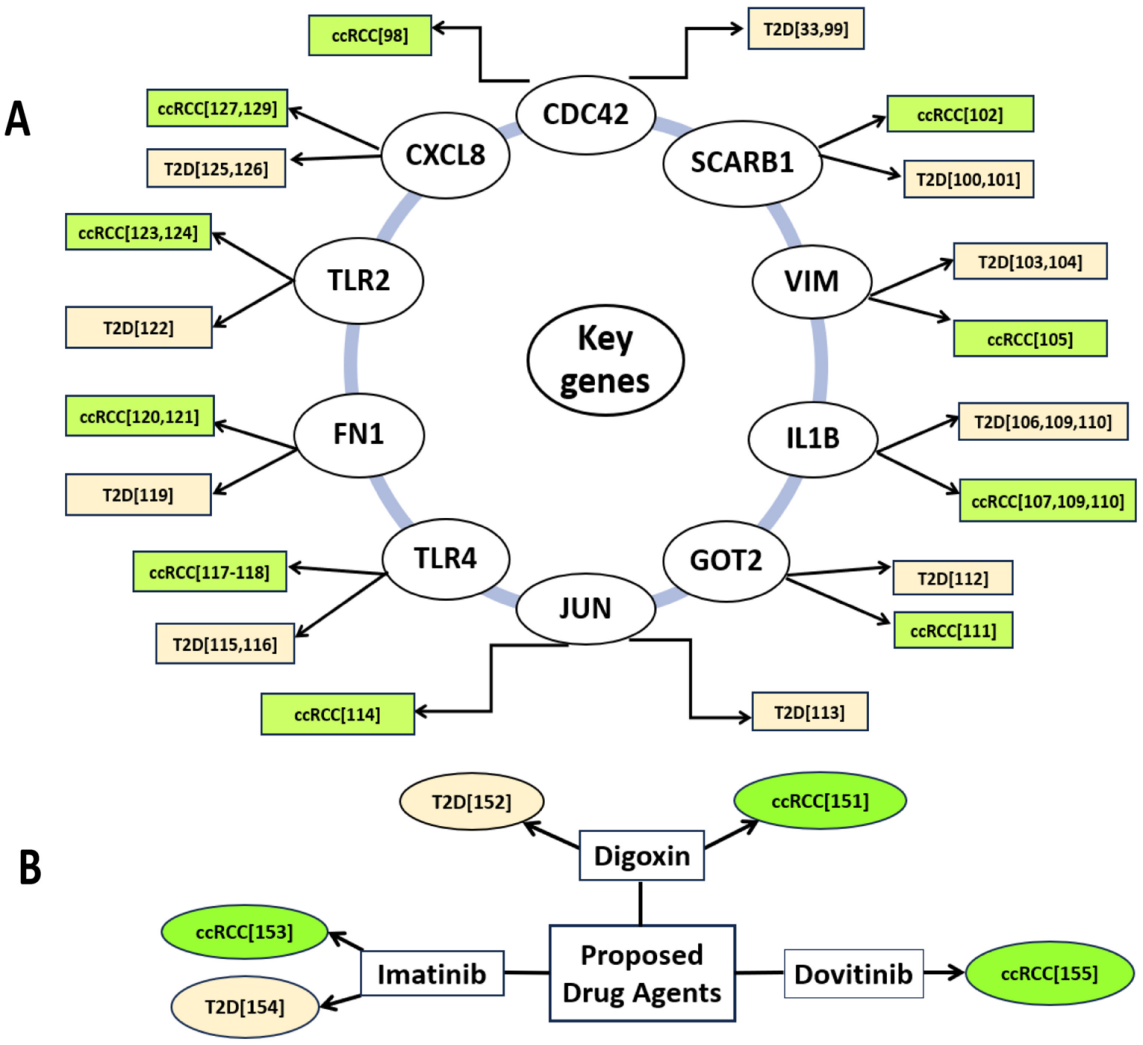
Verification of the proposed drug-targets (shared KGs) and drug-agents for T2D and CRC by the literature review. (**A**) Verification of the proposed drug-targets, (**B**) Verification of the proposed drug-agents.

Ttype-2 diabetes (T2D) is considered as one of the risk factors for clear-cell renal cell carcinoma (ccRCC)^96,97^. Therefore, identification of both diseases-causing shared key-genes (sKGs) is essential in order to investigate their common pathogenetic mechanisms and candidate drugs for better diagnosis and therapies during their co-occurrence. However, there was no study in the literature that has explored sKGs highlighting their pathogenic mechanisms, and candidate drug molecules as the common treatment for both T2D and ccRCC though diseases specific multiple drugs may create adverse side effects or toxicity to the patients due to drug-drug interaction^23–26^. In order to explore, common drugs as the representative of both disease specific multiple drugs, this study investigated the genetic relationship between T2D and ccRCC by detecting shared DEGs (sDEGs) that can separate both T2D and ccRCC patients from the control samples. We identified 259 sDEGs, where 194 upregulated and 65 downregulated. We computed local correlation coefficient between T2D and ccRCC based on the aLog_2_FC values of sDEGs by using Eq. 2, which is found as *r*_*XY*_ = 0.82, which indicates that both diseases are locally associated with each other through the expressions of sDEGs. Then we detected top-ranked 10 sDEGs (CDC42, SCARB1, GOT2, CXCL8, FN1, IL1B, JUN, TLR2, TLR4, and VIM) as sKGs (Fig. 2) for exploring their pathogenetic mechanisms and candidate common drugs for both diseases. The summary of this study are given in Figure S2 and the association of these 10 sKGs with both T2D and ccRCC also supported by some previous individual studies including CDC42^33,98,99^, SCARB1^100–102^, VIM^103–105^, IL1B^106–110^, GOT2^111,112^, JUN^113,114^, TLR4^115–118^, FN1^119–121^, TLR2^122–124^ and CXCL8^125–129^ as displayed in Fig. 6A. A study claimed that the gene ‘CDC42’ stimulates insulin secretion which is connected to the diabetes-related diseases, such as Diabetic Nephropathy(DN), ccRCC and various cancers^98^. Disorder of CDC42, can prevent healthy insulin secretion and promote diabetes. Most people agree that insulin resistance is the main factor for T2D^99^. As a result, CDC42 plays a significant role in the development of T2D, and treating T2D and associated disorders may benefit from targeted therapy for CDC42 ^33^. The polyligand membrane receptor protein SCARB1 involved in the glucose and lipid metabolism disturbance associated with T2D. A higher risk of T2D is linked to genetic variations of SCARB1^100,101^. Another study claimed that, SCARB1 is serve as both a therapeutic target and a diagnostic biomarker for ccRCC^102^. Vimentin, or VIM, has been identified as a key mediator of T2D linked to obesity^103,104^. On the other hand, VIM is considerably overexpressed in ccRCC cells^105^. According to the article GOT2 might be a useful prognostic indicator and therapeutic target for people with ccRCC^111^. Its expression in T2D and ccRCC is notably low^112^. A glycoprotein produced by fibronectin 1 (FN1) gene, is involved in host defenses, wound healing, blood coagulation, embryogenesis, and metastasis including other activities associated with cell adhesion. The up-regulation of FN1 is directly associated with the development of renal cell carcinoma (RCC)^121^ as well as DN^119^. The up-regulation of CXCL8 exhibits elevated serum levels in kidney cancer (KC) patients^128^ and early regulations in DN patients^129^. The protumor genes as well as RCC tumors are influenced by the up-regulation of IL1B gene^108^. Another two studies found the up-regulation of IL1B gene in T2D patients^109,110^. The expressions of TLR2 gene are found considerably higher in ccRCC tumor tissues^124^. Activation of TLR2 and TLR4 genes, and their overexpression are closely correlated with the severity of renal damage, according to a number of studies in different experimental models of kidney disease^130^. Creely et al. have found higher TLR2 expression in the adipose tissues of T2D patients^122^. In KC, renal inflammation and chronic fibrosis are significantly influenced by TLR4^118^. The long-term inflammatory condition cause of insulin resistance and the development of T2D^115^.

To explore transcriptional and post-transcriptional regulators of sKGs from TFs and miRNAs respectively, we performed sKGs-TFs and sKGs-miRNAs co-regulatory network analysis (Fig. 3) which detected five TFs proteins (YY1, FOXL1, FOXC1, NR2F1, and GATA2) and six miRNAs (hsa-mir-93-5p, hsa-mir-203a-3p, hsa-mir-204-5p, hsa-mir-335-5p, hsa-mir-26b-5p, and hsa-mir-1-3p) as the key regulators of sKGs. A transcriptional coregulator, Yin Yang 1 (YY1) stimulates the transcription of several long noncoding RNAs and its highly expressed in ccRCC^131^. YY1 in T2D^132^ and its role in the different symptoms as well as its interaction with signaling pathways that control the disease. Numerous disorders, including ccRCC ^133^, have been discovered to be regulated by the transcription factor known as YY1. FOXL1 and FOXC1 are members of the same family and perform similar functions. Thus, these genes might play significant roles in the ccRCC pathogenesis^134^. Another article shows that FOXL1 and FOXC1 are highly associated with T2D^135^.The development of ccRCC toward more aggressive molecular subtype is influenced by GATA2 proteins^136^. Another study reported that GATA2 is an important risk factor for T2D^137^, dyslipidemia and hypertension (HTN). Hsa-mir-335-5p has been shown to be involved in the management of RCC progression in a number of investigations^138^. Another article shows that, hsa-miR-335-5p appears to be associated in T2D by possibly influencing the expression of several candidate genes^139^. The Kaplan–Meier Plotter datasets show that miR-93-5p is highly expressed in ccRCC^140^.On the other hand, hsa-mir-93-5p which plays significant roles in post-transcriptional regulatory genes, especially in T2D^135^. Numerous kinds of cancer, including hepatocellular carcinoma (miR-9-5p), renal cell carcinoma (miR-1-3p)^141^, and thyroid cancer (miR-1-3p), may be impacted by the majority of the miRNAs examined. By examining the sKGs-set enrichment analysis, correlation of sKGs with distinct methylation of DNA, and regulatory analysis of sKGs using a variety of databases, we investigated the shared pathogenetic mechanism of ccRCC and T2D. We investigated critical biological processes (BP), molecular functions (MF), cellular components (CC), and KEGG pathways that are connected to the onset of T2D and ccRCC using enrichment analysis of the sKGs-set (Table 3). The ccRCC and T2D that were extensively enriched and caused important BP, MF, CC, and KEGG pathways were, Apoptotic process, inflammatory response, signal transduction, positive regulation of gene expression, identical protein binding, extracellular exosome, Lipid and atherosclerosis etc. these are disfunction and progression of T2D to ccRCC. Among them, Apoptosis, also known as programmed cell death, is the principal biological mechanism by which mammals destroy DNA-damaged cells and preserve tissue homeostasis. The failure of apoptosis increases the lifespan of tumor cells time and develops mutations, which can improve spreading during tumor cell development, improve tumor angiogenesis, and encourage cell proliferation (Fig. 1). Apoptosis is directly related to the control of T cells in ccRCC, and this must be considered in ccRCC immunotherapy^142^. Changes in signal transduction are always present in cancer such as, RCC. The dysregulated signal transduction that results from changes in proto-oncogenes and tumor suppressor genes ultimately promotes the abnormal development and proliferating of cancer cells^143^. In patients with T2D, hyperglycemia (also known as "glucose toxicity") may play a significant role in the development of insulin resistance and impaired signal transduction in the skeletal muscle^144^. Exosomes are crucial in the onset, detection, and management of kidney^145^, prostate, bladder, T2D^146^ and breast malignancies. The KEGG term, NOD-like receptors (NLRs) are widely used for pathogen identification receptors. NLRs play an important role in the cause of inflammation-induced insulin resistance(T2D), which leads to additional metabolic problems^147^. NLRs are divided into four subfamilies according to the type of N-terminal domains: NLRA, NLRB, NLRP, NLRC (C for CARD): NOD1, NOD2, NLRC3, NLRC4, NLRC5. Between, NOD1 and NOD2 inhibition has potential for treatment in acute kidney injury (AKI) ^148^. We also investigated using the DNA-methylation information with T2D and ccRCC shared KGs. An epigenetic process called DNA methylation involves adding a methyl group to cytosine bases, especially at CpG sites. Hypermethylation of CpG islands within promoter regions of tumor suppressor genes is widely recognized as a key mechanism leading to gene inactivation in various cancers^149^. In our investigation, we found that ten sKGs (CDC42, SCARB1, GOT2, CXCL8, FN1, IL1B, JUN, TLR2, TLR4, and VIM) were notably (*p-*value of < 0.001) within seven sKGs (SCARB1, FN1, IL1B, JUN, TLR2, TLR4, and VIM)) were hypomethylated at various CpG locations (Table S7). Therefore, it can be concluded that these ten hypomethylated sKGs have a substantial association with the growth and progression of ccRCC and the survival of the apoptotic process^150^.

In order to explore sKGs-guided common drug molecules for both T2D and CRC, we used molecular docking analysis and identified top-ranked four molecules (Digoxin, Imatinib and Dovitinib) that showed strong binding affinities with the sKGs-mediated target proteins. Then these molecules were verified for T2D and CRC by the literature review as displayed in Fig. 6B. To validate the drug molecules computationally, we conducted an ADME/T analysis and evaluated their drug-likeness. Two of the three medications that were shown to have drug-like properties were imatinib and dovitinib, which fit at least four of Lipinski's rule of five criteria. The chosen substances showed favorable ADME/T characteristics, possessing sufficient water solubility and high Human Intestinal Absorption (HIA) levels between 68.58% to 93.85%, and no carcinogenic effects. Among the top three identified candidate drugs molecules, Digoxin^151,152^ and Imatinib^153,154^ received support as the common candidate molecules for both T2D and ccRCC by the individual studies on T2D and ccRCC. It should be noted here that both drug molecules are already approved by FDA for the treatment of heart failure, atrial fibrillation (Digoxin), dermatofibrosarcoma protuberans, leukemias, systemic mastocytosis, myelodysplastic/myeloproliferative case, gastrointestinal stromal tumors and hyper eosinophilic syndrome (Imatinib), which can be found with Drug Bank (DB) accession ID DB00390 and DB00619, respectively. According to the reference article indicate that, Digoxin therapy significantly reduced cancer cell migration and proliferation in RCC cells and it is unique medicinal target for treating ccRCC patients^151^. It is claimed that, digoxin is a kind of cardiac glycoside that is used to treat heart failure as well as cardiac arrhythmias, that are both of common complication of T2D. Indeed, it is believed that up to 18% of diabetes patients receive digoxin^152^. In the animal model, imatinib effectively reduces blood sugar levels and treats the T2D^153^. Another experimental study reported that imatinib is a potent inhibitor against ccRCC^154^. The third proposed drug ‘dovitinib’ acts as an antagonist of some RCC-causing genes (VEGFR1, VEGFR2, VEGFR3, FGFR1, FGFR2, and FGFR3) according to an experimental study report^155^. Thus, we found that only Imatinib is experimentally validated in wet-lab for T2D and ccRCC individually, but not simultaneously. On the other hand, dovitinib molecule was experimentally validated with RCC only. However, the top-ranked drug molecule ‘Digoxin’ is not yet experimentally validated either with T2D or ccRCC though it is approved for other disease. Therefore, experimental validation is required for dovitinib molecule with T2D and the Digoxin molecule with both T2D and ccRCC. On the other hand, the proposed sKGs might be useful prognostic biomarkers in the development of immune therapy for ccRCC with T2D as discussed in different articles for other diseases^2,156,157^.

## Conclusion

This study detected ten shared key genes (CDC42, SCARB1, GOT2, CXCL8, FN1, IL1B, JUN, TLR2, TLR4, and VIM) that are able to differentiate both T2D and ccRCC patients from the control groups. The differential expression patterns of sKGs were also confirmed by some independent datasets from NCBI, TCGA and GTAx databases. Some significant shared biological processes, molecular roles, and pathways that are connected to the development of both T2D and ccRCC were identified by the shared key gene (sKGs) set enrichment analysis. The sKGs regulatory network analysis detected some TFs proteins and miRNAs as the transcriptional and post-transcriptional regulators of sKGs. The DNA methylation analysis detected some crucial hypo-methylated CpG sites that might stimulate the ccRCC development. Finally, sKGs-guided top-ranked three candidate drug agents (Digoxin, Imatinib, and Dovitinib) were discovered through molecular docking, drug-likeness and ADME/T analysis. The pipeline of this study might be a guideline to explore common pathogenetic processes and candidate drug molecules for taking a common treatment plan against other multiple diseases also. The output of this study might be potential inputs to the wet-lab researchers for further investigation in developing sKGs-guided effective common drugs against T2D and ccRCC.

### Supplementary Information

Supplementary Information.

## Data Availability

The datasets analyzed in this study are freely available at the following links https://www.ncbi.nlm.nih.gov/geo/query/acc.cgi?acc=GSE25724, https://www.ncbi.nlm.nih.gov/geo/query/acc.cgi?acc=GSE29226, https://www.ncbi.nlm.nih.gov/geo/query/acc.cgi?acc=GSE29231, https://www.ncbi.nlm.nih.gov/geo/query/acc.cgi?acc=GSE29221, https://www.ncbi.nlm.nih.gov/geo/query/acc.cgi?acc=GSE66270, https://www.ncbi.nlm.nih.gov/geo/query/acc.cgi?acc=GSE66272, https://www.ncbi.nlm.nih.gov/geo/query/acc.cgi?acc=GSE76351, https://www.ncbi.nlm.nih.gov/geo/query/acc.cgi?acc=GSE66271, https://www.ncbi.nlm.nih.gov/geo/query/acc.cgi?acc=GSE15932, https://www.ncbi.nlm.nih.gov/geo/query/acc.cgi?acc=GSE20966
