# Supplementary material for: Bioinformatics analysis to disclose shared molecular mechanisms between type-2 diabetes and clear-cell renal-cell carcinoma, and therapeutic indications
Source: Sci Rep. 2024 Aug 19;14:19133. doi: 10.1038/s41598-024-69302-w (PMC11333728; doi:10.1038/s41598-024-69302-w)
Supplement: Supplementary file 1 — Supplementary Information. [file 41598_2024_69302_MOESM1_ESM.docx]

**Supplementary File**

**Bioinformatics Analysis to Disclose Shared Molecular Mechanisms between Type-2 Diabetes** **and Clear-Cell Renal-Cell Carcinoma, and Therapeutic Indications**

Reaz Ahmmed^1^, Md. Bayazid Hossen^1,6^*,* Alvira Ajadee^1^, Sabkat Mahmud^1^*,* Md. Ahad Ali^1,3^, Md. Manir Hossain Mollah^4^, Md. Selim Reza^1,5^, Mohammad Amirul Islam^2^ and Md. Nurul Haque Mollah^1*^

^1^Bioinformatics Lab (Dry), Department of Statistics, University of Rajshahi, Rajshahi-6205, Bangladesh.

^2^Department of Biochemistry & Molecular Biology, University of Rajshahi, Rajshahi-6205, Bangladesh.

^3^Department of Chemistry, University of Rajshahi, Rajshahi-6205, Bangladesh.

^4^Department of Physical Sciences, Independent University, Bangladesh (IUB), Dhaka, Bangladesh

^5^Division of Biomedical Informatics and Genomics, School of Medicine, Tulane University, 1440 Canal St., RM 1621C, New Orleans, LA 70112, USA.

^6^Department of Agricultural and Applied Statistics, Bangladesh Agricultural University, Mymensingh-2202, Bangladesh

***Corresponding Author:** Md. Nurul Haque Mollah-E-mail: [mollah.stat.bio@ru.ac.bd](mailto:mollah.stat.bio@ru.ac.bd) (Bioinformatics Lab (Dry), Department of Statistics, University of Rajshahi, Rajshahi-6205, Bangladesh; Tel: +880-0721-711557)

| **Supporting Items/Captions** | **Pages** |
| --- | --- |
| **Supplementary Figures** |  |
| **Figure S1.** (A) Expression patterns of sKGs with Boxplots by GTEx and TCGA database (B) Boxplots with Independent T2D Data | **2** |
| **Figure S2.** The overview of this study | **3** |
| **Supplementary Table** |  |
| **Table S1.** Collection of T2D and ccRCC related candidate drugs from published articles and different online web-tools. | **3-5** |
| **Table S2.** List of upregulated and downregulated DEGs between ccRCC and control samples based four microarray gene expression datasets (GSE66270, GSE272, GSE76351, GSE66271) | **5-8** |
| **Table S3.** List of upregulated and downregulated DEGs between T2D and control samples based four microarray gene expression datasets (GSE25724, GSE29221, GSE29226, GSE29231) | **8-10** |
| **Table S4.** Common Genes are associated with T2D and ccRCC by statistical LIMMA approach | **10-11** |
| **Table S5.** List of upregulated and downregulated shared DEGs (sDEGs) between ccRCC and T2D | **11-12** |
| **Table S6.** List of shared key genes (sKGs) from PPI network based on different topological measures | **12** |
| **Table S7.** The significant prognostic value of CpG in sKGs | **13** |
| **Table S8.** Docking scores (binding affinities, kcal/mol) between the proposed receptors and top ordered 30 candidate drugs (out of 148) | **13-14** |

**Supplementary Figure**


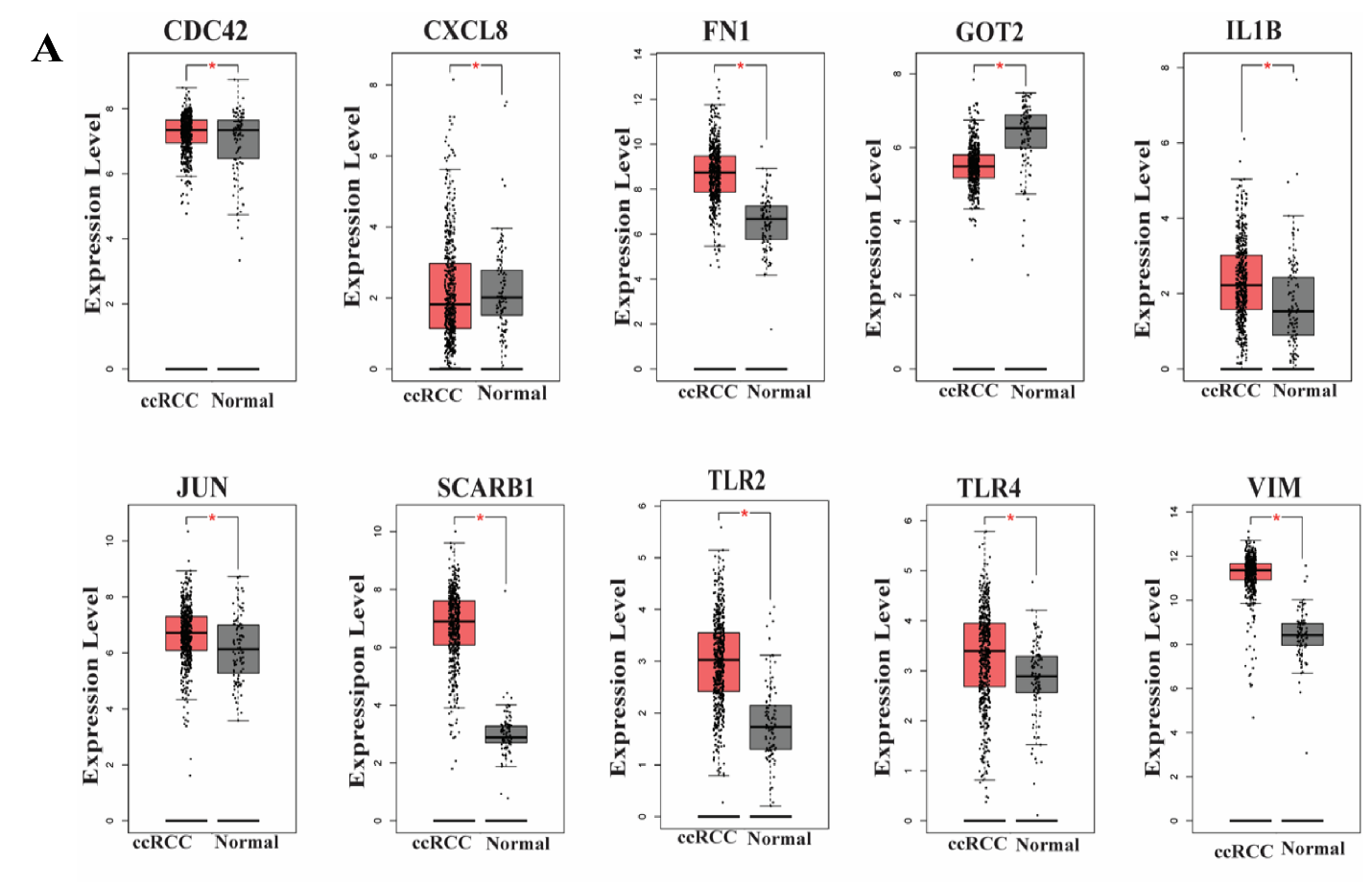


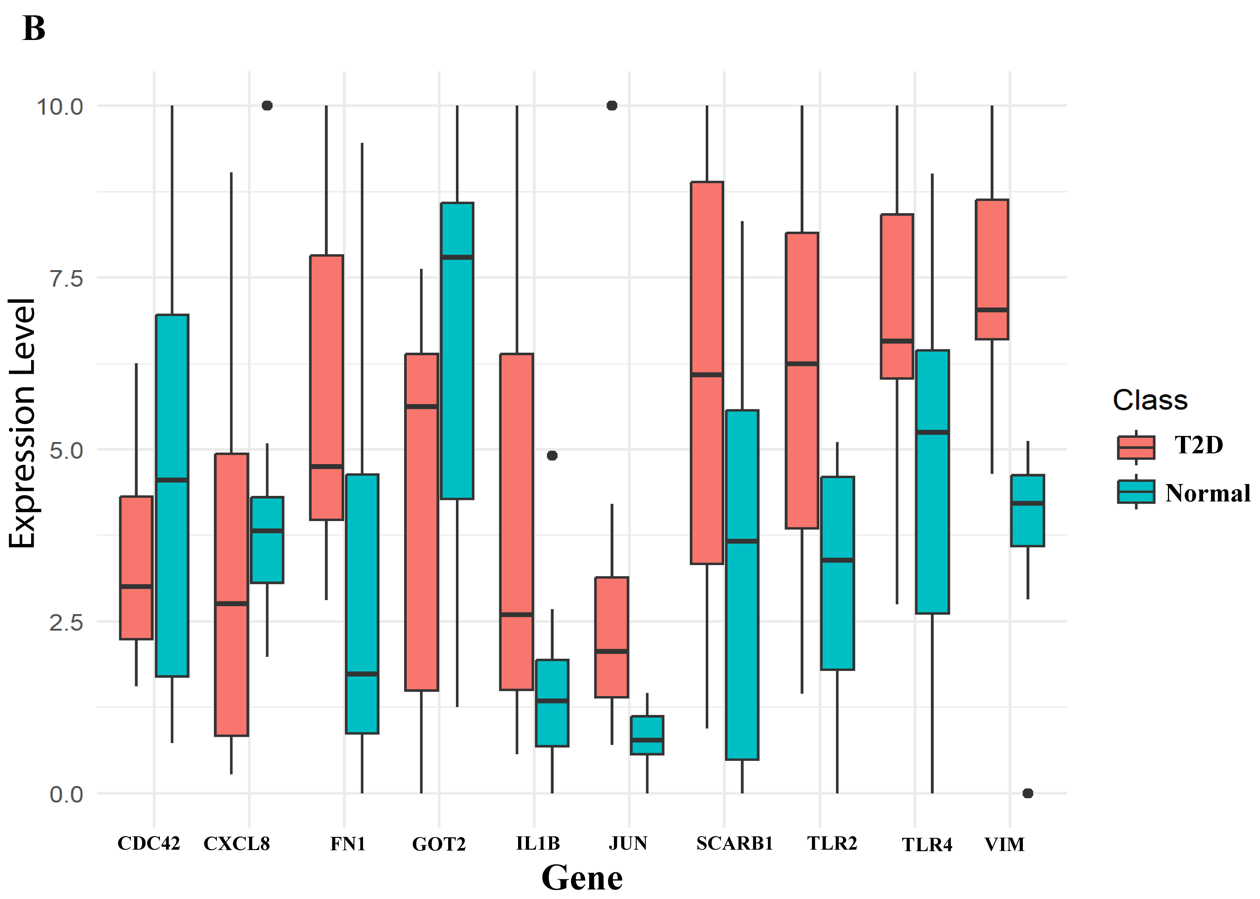


**Figure S1.** (A) Expression patterns of sKGs with Boxplots by GTEx and TCGA database (B) Boxplots with Independent T2D Data


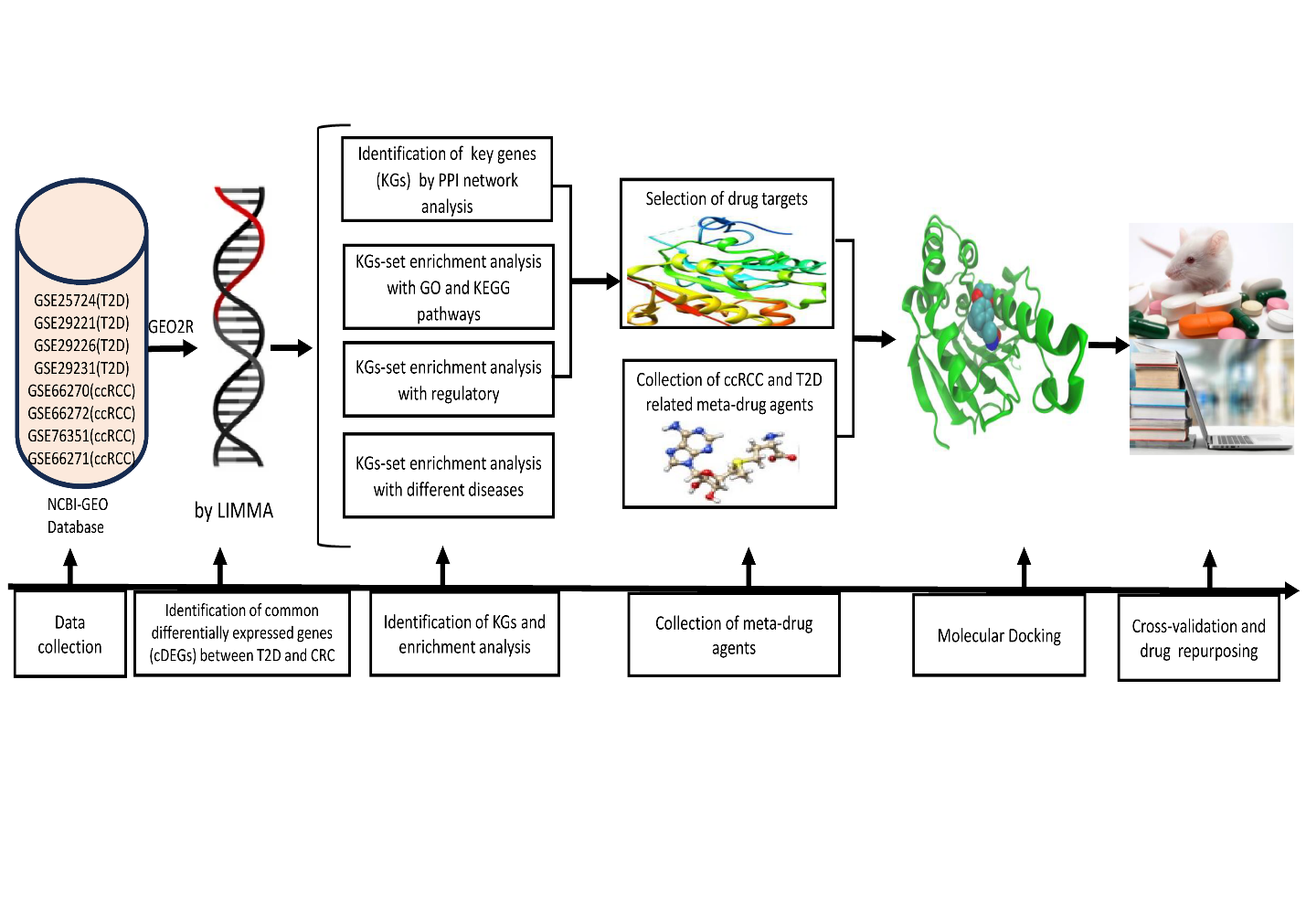


**Figure S2.** The overview of this study

**Supplementary Tables.**

**Table S1. Collection of T2D and ccRCC related candidate drugs from published articles and** **different online web-tools.**

| **Disease type** | **Paper title with reference** | **Drug list** |
| --- | --- | --- |
| T2D | Association of pharmaceutical teachers of India a study on prescribing pattern and potential drug-drug interactions in type 2 diabetes mellitus inpatients^1^ | Biguanides, Metformin, Glimepiride, Voglibose |
| T2D | Drug interactions of medications commonly used in diabetes^2^ | Sulfonylureas, Meglitanides, Exenatide, Thiazolidinediones (TZDs) |
| T2D | Low utilisation of diabetes medicines in Iran, despite their affordability (2000-2012): A time-series and benchmarking study^3^ | Glibenclamide, Gliclazide, Pioglitazone, Repaglinide, Acarbose, Chlorpropamide, Sitagliptin, Pioglitazone |
| T2D | Structure Based Drug Designing for Diabetes Mellitus^4^ | Acetohexamide, Phenformin, Miglitol, Tolazamide, Voglibose |
| T2D | Multiple therapeutic effect of endothelial progenitor cell regulated by drugs in diabetes and diabetes related disorder^5^ | Vildagliptin, Amlodipine, Aliskiren, Simvastatin |
| T2D | Integration of the Drug-Gene Interaction Database (DGIdb 4.0) with open crowdsource efforts^6^ | Canakinumab, Rilonacept, Tt-301, Tiludronic Acid, Risedronic Acid, Pentamidine, Thyroglobulin, Mafosfamide, Lansoprazole  , Rabeprazole, Ocriplasmin, Bruceantin, Sergeolide, Irisolidone, Neochamaejasmin A, Chembl477052, Holacanthone, Sangivamycin, Retinylretinoate, Ciprofibrate  Ranibizumab, Pegaptanib Sodium, Bevasiranib, Elmiron, Aflibercept, Bevacizumab, Cdc-801, Sildenafil, Cilostazol, Sunitinib, Resveratrol Hexanoic Acid, Eritoran, Nelfinavir, Saquinavir, Infliximab, Pravastatin, Methotrexate, Tacrolimus, Foscarnet, Cidofovir, Pamidronic Acid , Hydroquinone, Canertinib, Ionomycin, Methimazole, Leflunomide, Naproxen, Ceftriaxone, Lansoprazole, Sunitinib, Clarithromycin, Maraviroc, Leronlimab, Vicriviroc, Pexidartinib, Vatalanib, Sunitinib, Imatinib, Sorafenib,Vemurafenib, Dovitinib, Alteplase, Erythromycin, Pravastatin, Pentoxifylline, Verapamil, Cefaclor, Nicardipine, Omeprazole, Hydroquinone, Infliximab |
| T2D | δ-Aminolevulinic acid-induced fluorescence unmasks biological intratumoral heterogeneity within histologically homogeneous areas of malignant gliomas^7^ | Clobetasol Propionate, Mercuric Chloride, Ofloxacin, Meloxicam, Emetine, Sulfaphenazole, 6-Mercaptopurine, Amitriptyline, Beta-Estradiol 3-Benzoate, Busulfan, Thioguanine, Beta-Estradiol, Clofibrate, Chlorambucil, Digoxin, Indomethacin, Atorvastatin, Bisphenol A, Chloroxylenol, Doxorubicin, Rosiglitazone, Oxybutynin, Pralidoxime Chloride, Zidovudine, Mesna, Mebendazole, Nifedipine, Gemfibrozil, Phenacemide, Ampiroxicam, Fluphenazine, Flavoxate, Candesartan, 4-Nonylphenol, Etoposide, Streptozotocin, Myrtecaine, Venlafaxine, NN-Dimethylformamide, Norethindrone, Propylene Glycol, Benzethonium Chloride, Crotamiton, Daunorubicin, Doxifluridine, Aspirin, Buflomedil, Clonazepam, Etoposide, Trichloroethylene, Carmustine, Bithionol, Dactinomycin, Harringtonine, Nitrendipine, Praziquantel, Tetracycline, Tocainide, Azathioprine, Bis(2-Ethylhexyl)Phthalate, Epirubicin, Rofecoxib, Clonidine, 1-Naphthyl Isothiocyanate, 2-Acetylaminofluorene, Carboplatin, Clomiphene, Chlordiazepoxide, Oxyquinoline, Dexchlorpheniramine, Mitomycin, Ciprofloxacin, Captopril, Cefotaxime, Fenoprofen, Fluvastatin, Sulfadoxine |
| ccRCC | Mechanisms of Acquired Resistance to Tyrosine Kinase Inhibitors in Clear - Cell Renal Cell Carcinoma (ccRCC)^8^ | Sunitinib, pazopanib, tivozanib, sorafenib, axitinib |
| ccRCC | As a library, NLM provides access to scientific literature. Inclusion in an NLM database does not imply endorsement of, or agreement with, the contents by NLM or the National Institutes of Health^9^ | Pazopanib, Cabozantinib, Everolimus, Temsirolimus, Nivolumab, |
| ccRCC | Current and emerging therapies for first-line treatment of metastatic clear cell renal cell carcinoma ^10^ | Nivolumab, atezolizumab, Bevacizumab, pilimumab |
| ccRCC | Metastatic non–clear cell renal cell carcinoma treated with targeted therapy agents: Characterization of survival outcome and application of the International mRCC Database Consortium criteria^11^ | Everolimus, Temsirolimus, Tivozanib, Pazopanib, Bevacizumab, Axitinib |

**Table S2. List of upregulated and downregulated DEGs between ccRCC and control samples based four microarray gene expression datasets (GSE66270, GSE272, GSE76351, GSE66271)**

| **Downregulated cDEGs** | **Upregulated cDEGs** |
| --- | --- |
| ZC3H14, SLC16A10, CISH, CLIC5, LIN52, HPCAL4, COA3, FBLN5, NR3C2, NT5E, NAPEPLD, ABCB1, PTPN13, LOC100506870, FAM171A1, PTPN4, TTC39B, PSMA5, SCD5, PTCH1, CRLS1, B3GALT2, AGFG2, DAB1, ACSL6, MINOS1, CYP2E1, PATJ, SOX13, PRSS35, PURA, TAMM41, MEST, HERC4, RUNX2, CHRM3-AS2, STK38, ELOVL4, WDR86-AS1 | NDUFA4L2, CA9,EGLN3, IGFBP3, BHLHE41, SAP30, SPAG4, PHKA2, QRFPR, DDB2, SNORA64, CD300A, PFKP, NETO2, LPCAT1, FCGR3B, PPP1R3B, LAPTM5, ARHGDIB, CXCR4, C1QC, HILPDA, ZNF395, GMFG, TLR8, BIN2, EHD2, TNFAIP6, CASP1, SEMA5B, PML, IKBIP, VIM, HCLS1, FLI1, RASSF2, LGALS1, PAG1, PLK2, VEGFA, LAIR1, APOC1, C1orf162, IFI16, LCP2, TMSB10, ETS1, MS4A6A, C1QA, C1QB, RNF145, PSMB9, EVI2B, FAM57A, CTSS, PDK1, DPYSL2, MNDA, SCD, P2RX7, HLA-F, ZEB2, PTPRC CAV2, ENO2, VAV1, KIAA0101, CYBB,nHLA-DPA1, PYGL, PTRF, PTPRE, SLC15A4 LYZ, CD53, LY86, MLKL, OSMR, HLA-DRA, STAMBPL1, RNASET2, ITGB2, MSR1, PLEK, EGFR, CAV1, RGS1, EVI2A, MARCKS, CX3CR1, TIMP1, FN1, NUSAP1, RRM2, GPR34, APOBEC3C, GIT2, ARHGEF10, SRGN, CD86, PCDH17, ENTPD1, PTPN12, PRR11, PECAM1, DOCK10, LOXL2, PDLIM1, PIK3R5, ELF4, PRDM1, CLEC7A, MEF2C, YWHAH GNB4, SH2B3, SACS, MPEG1, ARHGAP30 OGFRL1, MAP4K4, TRIM22, MYC, TNFAIP3, CXCL16, ITGAL FLT1, FPR3, TMEM45A, NKG7, FABP6, CSF1R, ARHGAP25, MYADM, PROCR, ASAP1,TAGAP, TREM2, BIRC3, IL10RB, TCF4, 1-Mar, CD74, CD37, CCND1, GGTA1P, MS4A4A, RCN1, ICAM1, CD52, CLEC4A, ADGRE5, C3, PMP22, SPARC, TUBB6, TGFB1, FCGR2A, RAP2B, ZFAS1, RCSD1, FAM198B, RASSF3, SLAMF7, LAMA4, RHOH, ALDOC, ABCG1, BTK, RAC2, CD84, C10orf10, CORO1C, CCNA2, HCK, IGSF6, LST1, PARVG, SETD7 FAS, NCK1, AXL, DTL, PRKCDBP, UBE2L6, AHNAK2, FCGR1CP, TAPBP, LCP1, TAP1, GIMAP2, ALOX5AP, TUBA1A, APOBEC3G, INPP5D, NCKAP1L, CPNE2, WIPF1, FAM129A, BAZ1A, IL4R, CD48, GJC1, RGS18, SLC1A4, RASSF5, GLIPR2, CENPU, CORO1A, IKZF1, TMEM173, TPM1, COLGALT1, CSF2RA, GJA1, SLC43A3, ELMO1, LILRB2, CD14, CXCL10, CTHRC1, ADCY3, TLR2, PLXDC1, ZAK, GNG2, SERPINH1, PREX1, CCL5, CD99, S100A10, CLIC2, GAPT, MYOF, C3AR1, PIK3CG, TYMS, IL10RA, HLA-DQB1, ADCY7, NCF4, PPP1R18, MCAM, CARD16, CD1D, CCDC88A, BCL6, FAM49A, LY96, PLIN2, VSIG4, ARPC1B, CDCA7L FAR2, RGS5, LYN, PPP1R13L, PLEKHG2, THEMIS2, TOP2A, CD4, EZH2, NRP2, ADM, TNFRSF10B, ITGA5, SLC27A3, NPTX2, P2RY13, CBFB, MCM5, PKM, FLNA, RNASE6, PLOD2, ANGPT2, COL4A2, GBP2, PGM2L1, TNFSF13B, CHST15, RAB31, ANXA2, TGFBI, GPR65, NFE2L3, PPP1R3C, TES, PLAC8, RND3, TM6SF1, CD180 YBX3, TLR4, LHFPL2, VCAN, HHEX, KCNJ2, RHBDF2, LILRB1 ADGRE2, HLA-DMB, ALOX5, COL4A1, IRX3, CLEC2B, PRF1, AFF3, PLXND1, TLR7, SCARB1, ASPM, NNMT, SLC1A3, DENND5A, SLFN5, PRELID2, GZMA, CHST11, COL1A2, HAPLN1, EMILIN2, EMP3, LDLRAD3, C1S, GAS2L3, ARL4C, TBXAS1, TAGLN2, VWF, OAS3, CEP55, MET, P2RY1, AFAP1L1, FMNL3, PYCARD, SLFN11, TPX2, SEC24D, MYBL1, ZC3HAV1L, EPB41L2, RNF125, CCND2, CXCL9, SLFN13, KCNMA1, SELPLG, SLA, CD33, CD93, CALU, RTP4, TRPV2, DNMT1, GNLY, HLA-DPB1, PMEPA1, WWC3, STK17B, STK10, ADGRL4, CD44, VASH1, MAP3K12, BTN3A1, MFNG, DIAPH2, ELK3, CMTM3, CP, CD163, COL21A1, SPI1, GRAMD4, DCLK1, TNFSF8, PTP4A3, CD247, SAMD9, ARRDC2, APBB1IP, SMC4, PGBD5, ACKR3, EMB, TCIRG1, DOCK2, AOAH, CXCL11, TNFRSF1B, SP140L, CALHM2, ANLN, CCL18, CDH13, CST7, LSP1, MSC, CXorf36, HEY1, MYO1F, JAZF1, APOLD1, ITGAX, LAMC1, UHRF1, IFI44, NLRC5, CSF2RB, BMP1, KLHL6, PLEKHO1, AKNA, ACTN1, CSF3R, SIGLEC10, IDO1, TGM2, CD3D, EOGT, ITGAM, GZMK, PRC1, CARD8, ARHGEF6, C18orf54, CD36, NCF2, SEMA4C, ADA, SHC1, OAS2, PTPN22, OLFML2B, CCL28, FYB, ANXA1, ITPRIPL2, LRP1, CYTH4, GBP4, B3GNT5, HLX, ITK, TMEM154, CFD, CAMK1D, STAB1, LRRC25, BUB1, ADAP2, ANGPTL2, CLEC5A, PTGS1, COL3A1, DDIT4, CD70, LOX, SIPA1L2, IL27RA, SYT11, COL15A1, FPR1, CELF2, IFI44L, CEBPB, CD2, TIMP2, CLEC12A, NFATC2, KCNE4, ATAD2, FRMD6, BTN3A2, ASF1B, SERPINE1, FCGR2B, SH3RF3, ATP2B2, IL2RG, KCNK3, TRPA1, COL6A2, MARVELD1, IL3RA, SERPINB9, PGF, LRRK1, COL8A1, XAF1, MAP3K7CL, DGKA, PALD1, FSCN1, HRH1, IL2RB, CASP4, SLFN12, CENPK, ARL4A, CDH11, GRK5, STC2, COL5A2, CD69, TP53, PXDN, MIS18BP1, PIEZO2, ITPR3, CLEC2D, KCNS3, CDKN3, ANO4, CDC6, CCNE2, SERPINB8 , LAYN, FKBP10, COL1A1, LPXN, LAMP3, GDPD5, IFI27, TNFSF9, MMP14, PNCK, FBN1, GPR183, DUSP1, DUSP5P1, CRIP1, GBP5, GRIK3, CDKN1A, CNRIP1, HIST1H2BK, SLC2A3, PUS7, ANOS1, SDC3, MIR155, DOK2, ACTG2, GPX8, ABCA12, CLIP2, BCAT1, PDLIM3, BCL11A, PCDHB14, ADAMTS2, CDH4, JAML, CCNB2, CDON, MYO1G, MAP3K6, TPM4, ADAM15, WDR76, BASP1, AHR, COL6A3, CENPF, SEMA3F, DUSP10, FAP, FERMT3, MILR1, CYGB, GPR176, MYO9B, C3orf70, CSPG4, RSAD2, SMOX, 3-Mar, 9-Sep, GABRD, PLAUR, FBLN2, MKI67, EDNRA, LAMA3, RHOJ, SEZ6L2, FGD2, GINS2, PTPRCAP, S100A11, PLP2 DOK1, NLRC4, TM4SF1, APOL1, OLFML2A, TNFRSF9, GXYLT2, PCDHB18P, IL7R, RASA3, CD300C, THBD, CCDC102B, LFNG, MB21D1, SAMSN1, ADAM12, GNG11, RRAD, FOXM1, TSPAN13, ARNTL2, DKK3, MCTP1, SLC7A11, EHD4, COL5A1, PMAIP1, KIAA1462, CD276, KCNAB1, C1R, NOV, TRIO, CPE, BEST1, EPHA3, TLE1, NRROS, SLC2A1, NAIP, TNFRSF12A, EMP1, CSF1, APCDD1, HGF, FMNL1, PREX2, PRR16, DPYSL3, IKZF3, TREM1, GPR85, EDIL3, TSHZ2, PADI1, THBS2, AREG, AQP9, PLCB4, ADAMTS12, KCNJ8, MDK, SLC11A1, POSTN, ELOVL2, SRPX2, ARHGEF2, PRRX1, ESCO2 |

**Table S3. List of upregulated and downregulated DEGs between T2D and control samples based four microarray gene expression datasets (GSE25724, GSE29221, GSE29226, GSE29231)**

| **Downregulated cDEGs** | **Upregulated cDEGs** |
| --- | --- |
| PRDX4, SEC62, KDELR2, ARMCX1, ZFAND1, TMX1, ZNHIT6, EEF1A1, ARPP19, CPNE3, AKAP11, TMEM50A, PEF1, FAM208A, PGRMC1, SERPINI1, SEC23B, SNX2, ENPP2, UFM1, ADSS, ANKRD49, SERINC3, OSTM1, CFAP20, WNK1, RFC5, SLBP, RAP1GDS1, LEPROT, YWHAQ, KCTD3, TVP23B, ALDH9A1, CNIH1, TMEM208, PCBP2, TGFBR2, OAT, CDK4, CD99, RTN1, CLN5, ADIPOR2, TMED3, PDCD10, ALG9, MAN1A1, COPB2, POGK, GTF2E2, APLP2, AP3B1, TMEM30B, CANX, ICMT, LETMD1, CREB3L2, FTL, TCTN3, PSMG2, TRIM13, GCA, KIF2A, NDEL1, HSD17B11, EXOC2, ATP6AP2, SEC61A1, ZNF207, VPS4B, TRAM1, LIPA, PIGP, SC5D, GLUD1, CKS1B, ITGB1BP1, TROVE2, PIGC, PWP1, ERP44, RNF38, PPCS, NET1, NUP160, ACLY, KCTD9, MXRA7, KIF1B, RABAC1, EBAG9, LRRC8B, DUSP12, PDCD6IP, VIM, DTWD1, ARF3, DEGS1, GUCY1B3, COMMD8, ZNF451, CTSC, RBM12, ZFP36L2, ASNS, APMAP, GOLGA7, GNS, FAIM, TRIP4, DEF8, CDC14B, DENND5A, GLG1, GOLPH3, PON2, ORC5, PHACTR2, HNRNPA3, GRAMD3, KRT10, WASF3, RAB22A, SS18, MARK1, ATP6V0E1, TMEM87A, TMEM50B, IFT52, CTBS, PDCD6 ITPR1, CCT6B, MAP1B, NECTIN3, PIP4K2A, SH3BGRL, TFPI, PEMT, PKD2, EHBP1, SNX17, TMEM14A, SLC35A1, DNMT1, EPS15, ID3, SLC35A5, CBX1, CHPT1, PLPP1, ITM2B, ALDH18A1, MAPK9, TRAK2, SPTSSA, TRAK1, PIAS1, BMP2, CHN1, MAOA, APTX, PARP4, MED8, SWAP70, SNRK, SCPEP1, RDH11, DSE, FERMT2, CLTA, PURA, JADE3, PRPSAP1, PDGFRL, APP, CETN2, HMGN1, SSR2, CCND2, RBMS1, POLR1D, BAG5, TM9SF3, PRCP, DPY19L4, SMC2, RBPJ, CD9, SCD, PLCB4, BCAP29, STXBP6, FAM3C DYNC1I2, FTH1, FRYL, RNH1, SAP30BP, USP3, TGFBR3 PPP2R2B, SERPINB1, HEBP1, TRMT13, SDCBP, JAK1, SRI, PGAM1, TCEAL9, YWHAB, CYB561, USP8, DPYSL2, ITGAV, DSTN, CDC42, KCTD12, NEU1, ATP2C1, ZNF415, FAM206A, ACOT9, LGALS8, HNRNPR, PELI2, NR2F2, UGGT2, TMEM123, TCEAL2, BTG1, IFT57, MAPKAPK5, CD58, ANKRA2, ABCG1, EXT2, NPC2, TRPC1, GNG10, PLA2G16, BCKDHB, PRMT2, EOGT, PDCD2, TMEM243, CCNC | COL7A1, FHL1, CLTCL1, PLEKHF1, NR4A3, BHLHE40, MYH3, MED24, GRB10, MYBPH, EXOC7, TAPT1, ACTN2, MFN2, PDLIM7, GOLIM4, CLIC5, TGFB3, CASQ2, HCFC1R1, MRPL41, TMEM161A, HES1, SLPI, PTP4A3, TRIP10, RAPGEFL1, AIMP2, RORC, BTG2, MYOM1, LONRF1, ICOSLG, DMD, YBX3, MCEMP1, TLR2, HP, LILRA5, SLC2A3, S100A12, BST1, SIGLEC5, CLEC4D, IRAK3, LIN7A, CD177, CNIH4, GPR84, KREMEN1, MMP9, ARG1, ANKRD22, VNN1, DYSF, TIMP2, ANXA3, SOCS3, BMX, KIF1B, CYSTM1, FCER1G, PFKFB3, MCTP1, ADM, TLR4, RAB20, FCGR1B, PLBD1, SERPINA1, FKBP5, SIPA1L2, GADD45B, ETS2, OSM,FN1, SIRPB1, SORT1, JUNB, TLR2, SERPINB8, FOSL2, IL1B, PGD, NFKBIA, HK3, DIRC2, MANSC1, FCGR1A, ELL2, PHC2, LRG1, GNA15, MAFG, GLT1D1, RAB32, CSTA, OSCAR, PDZD8, MGAM, NFIL3, ARHGAP24, BASP1, ADGRG3, NBN, FCAR, BCL2A1,CXCL8, SLPI, PYGL, CYP1B1, SMPDL3A, SAMSN1, GADD45A, ACSL1, PADI2, TRIB1, IER3, SLC22A4, JUN, FBN2, SIRPA, RNF144B, RBM47, FGD4, CDA, PLAUR, 1-Mar, CD55, CXCL16, JMJD6, AQP9, GAS7, KCNJ15, ABCA1, BCL6, LILRB3, CLEC4E, CEACAM8, TNFAIP6, IL1R2, WDFY3, TLR4, ORM1, CD163, CEBPD, LILRB2, FOXC1, GLUL, MS4A4A, PADI4, DSC2, IL1RN, ASGR2, CCRL2, PDGFC, TLR8, MEGF9, S100P, G0S2, ADAM9, LPCAT2, EPAS1, QPCT, TFPI, STEAP4, OLR1, ICAM1, IRS2, PPP1R3B, C15orf48, PROK2, PROS1, LILRA3, NAMPT, LRRK2, CD36, SSFA2, FPR2, TMTC1, FOLR3, FCGR2A, SLC7A5, FAM198B, PAQR8, LY9, TSPAN5, C1orf74, CCR2, ANKEF1, YME1L1, ZMYM6, CISH, GCSAM, ZNF578, CCR5, TMEM35B, LOC283588, DENND2D, WDR86-AS1, THAP2, VPS45, GIMAP1, GIMAP7, POLR2H, GEMIN6, TMEM204, RORC, MRPS30, DUSP7, TRAF3IP2-AS1, POLH, ICMT, MRPL47, POLR1B GIMAP1, PDE7A, NT5E, ARL10, UBE2T, GPALPP1, COG6, THNSL1, GIMAP6, LOC153682, RAD54B, ASF1A, ELOVL4, NUP43, C17orf80, NUDT5, CLIC5, CCDC65, NMT2, GZMA, TDRKH, SLC16A10, ERCC6L2, MCPH1-AS1, DNAJC24, PITPNC1, GMPS, SYNRG, ICAM2, HSD11B1, MSC-AS1, IGIP, GBP4, APOBEC3G, HPCAL4, UBXN10-AS1, MCM9, KCTD7, RAB22A, TTC39B, STX17, MGC40069, KBTBD3, FANCF, TRG-AS1, B3GALT2, LOC100128751, OPTN, HMG20A, TNFAIP8L2, SLAMF1, TBC1D31, XIST, TARP, GIMAP4, PTCH1, HPF1, ST8SIA1, ZNF264 VSIG1, LOC100506870, NIF3L1, NEFL, GALNT12, HOPX, THYN1, NSG1, AMIGO1, ZNF202, PREPL, ZADH2, WDCP, TRMT1L, METTL21B, RNASEL, TRAF3IP3, PRKCQ-AS1, NHSL2, SOX13, NCR3, ACSL6, SLC35B3, LOC101928673, CFH, VPS72, ODF2L, ZNF426, LOC100132057, UBASH3A, LYRM7, FBLN5, TSIX, AGFG2, PURA, ZNF137P, ZNF512, C9orf85, TAGAP, GIMAP8, SEC23IP, SLFN5, INPP4A, RUNX2, CAMTA1, ZNF248, LINC01260, LIN52, PTPN13, UBE2L3, ZFP3, RNF144A, MBLAC2, HN1L, SPON1, TGFBR1, TYSND1, RBL1, DAB1, LPAR5, SEC62, ZNF792, LINC00892, CREBL2, KLHL6, ZNF320, ZNF544, KIAA0040, CRLS1, CRTAM, PATJ, AMIGO2, DFFB, TSPAN18, LOC283357, 6-Sep, THEMIS, TRIM13, RNASEH2B, PET117, ITGA4, DCLRE1C, TMEM60, DDX58, LSM11, FLJ32255, CYB5A, NAPEPLD, SERPINI1, C10orf128, RPAP2, ZNF420, SATB1, GIN1, MINOS1, GVINP1, MGAT2, MRPS14, BCL2L13, AQR, CYB5B, ARV1, DLG3, KDSR, NTPCR, SIRPG, C18orf54, SLAIN2, C2orf42, ERGIC2, INTS7, HERC4, ZNF260, ZKSCAN7, ZNRD1ASP, HNRNPR, ZBTB3, SNAPC5, NMRK1, HCG18ZNF148, NFYB |

| **Table S4. Common Genes are associated with T2D and ccRCC by statistical LIMMA approach** |
| --- |
| PRDX4, VIM, VEGFA, APOC1, DPYSL2, SCD, CAV2, IFNAR2, ACLY, LIPA, ENTPD1, TMEM87A, BCAP29, ABCG1, SERPINB1, AXL, TRADD, DEGS1, ADCY3, CCL5, CD99, MYOF, FHL1, GUCY1B3, DNASE2, PRKCSH, SLC38A1, USO1, SAP30BP, YBX3, SERINC3, FRYL, KCTD9, SCARB1, DENND5A, SLC35E1, ALDH18A1, CCND2, CDK19, KCNMA1, SIPA1, TMEM243, DNMT1, PMEPA1, CASP2, BTG1, CKS1B, PTP4A3, KCTD3, PRMT2, SRI, TCIRG1, JAK1, SWAP70, CANX, RUNX3, SSR2, SP110, UNC93B1, ME2, POLR1D, CD3D, EOGT, TMEM123, CALD1, NCOR2, TBCE, PIGC, PRSS23, TNFRSF14, RNF34, HLX, YWHAQ, TRAM1, CAMK1D, LMNA, GZMH, PWP1, LGALS8, CD2, TIMP2, TRIP10, BMP2, CD58, TMX1, KIF2A, PPP3CC, EXOC7, RFC5, GRK5, FAM13A, ZNHIT6, RBPJ, PDCD6, PXDN, GAS7, RBM12, TRPC1, EHBP1, TMEM50A, DUSP12, PGAM1, PLPP1, ADNP, KDELR2, TCP11L1, PQLC3, ADAM15, PON2, DTWD1, ZNF207, S100A4, RNF14, ENG, PDLIM5, MED14, TMED3, EXT2, HCFC1R1, MAPK9, LYL1, TCF3, ASNS, RBMS3, UFM1, POLG, RNH1, LLGL1, DSE, PIP4K2A, RASA3, NUP160, CDKN2C, RCC1, SDCBP, SNX2, TRANK1, RAP1GDS1, PLXNA3, VWA1, CAPRIN1, GRAMD3, CXorf57, INF2, SF3A2, ZFAND1, RTN1, KIF1B, ICOSLG, ID3, CTSC, PLEKHF1, THBS1, NOD1, LRCH4, SNRK, BHLHE40, AKAP13, ITGB4, PLEKHA4, UGGT2, SEC61A1, DMD, CDC42, RARRES1, TMEM209, DPY19L4, THBS2, IFRD1, KCTD12, CHN1, ORC5, CD9, PLCB4, RRBP1, OSTM1, PIK3IP1, ADGRA2, MAP1B, EFEMP2, PGRMC1, MAP4K1, TFPI, MFN2, RAB6B, CD82, WISP2, GPD1L, ALDH6A1, SPHK2, TCEAL2, TMEM30B, WNK1, ECI2, RDH11, CLIC5, LHPP, SCAMP4, TRAK1, PELI2, KTN1, AHCY, SMARCC1, CDC14B, CDKN1C, GOT2, PDCD6IP, PCBP2, OAT, ICMT, WASF3, SPOCK2, TGFBR3, ACADM, UQCRFS1, SC5D, PPP2R2B, BCKDHB, DNAJC12, CLCN7, COL7A1, SPTSSA, ALDH1A2, ALDH9A1, MPRIP, LONRF1, RPS29, APOO, ATN1, NR2F2, METTL7A, ADAP1, ATP1B2, PTK2, MRPL41, TTC38, GAK, CTTN, LRFN3, CYB561, GOT1, CTIF, PDE7B, IFT57, DMWD, GLG1, MARK1, TSKU, MAOA, CA2, COMMD8, GLI3, SNTB2, CREB3L2, CHPT1, TMED5, TMEM50B, APLP2, MAN1A1, ITPK1, LIME1, ATP5L, NDUFAF4, DHRS7, MLXIPL, FBLN1, ATP6V0E1, GAS8, NUDT3, SNAPC4, SLC25A11, AGRN, CTSB, FERMT2, GOLIM4, TAOK2, COX5A, ITPR1, GLUD1, DLG4, EXD3 RORC, PURA, ACTN2, SOGA1, GPR137, SCPEP1, WNT11, TSPYL1, LIPE, TRMT13, BTG2, ISLR, PDSS1, APOE, AKAP11, COPB2, ACOT7, PHLDB1, ZNF451, CCNC, TAPT1, SETD1A, CLTA, ATP2A2, DDX17, EML3, SMG9, ARHGAP33, LEPROT |

**Table S5. List of upregulated and downregulated shared DEGs (sDEGs) between ccRCC and T2D**

| **Downregulated sDEGs** | **Upregulated sDEGs** |
| --- | --- |
| TCEAL2, TMEM30B, WNK1, RDH11, TRAK1, PELI2, CDC14B, PDCD6IP, PCBP2, OAT, ICMT, WASF3, TGFBR3, SC5D, PPP2R2B, BCKDHB, SPTSSA, ALDH9A1, BCAP29,CDC42, NR2F2, UGGT2, CYB561, IFT57, GLG1, MARK1, MAOA, COMMD8, CREB3L2, CHPT1, TMEM50B, APLP2, MAN1A1, ZNF207, KIF1B, CTSC, ATP6V0E1, FERMT2, ITPR1, GLUD1, PURA, SCPEP1, DTWD1, TRMT13, CXCL8, TMED3,GOT2, AKAP11, COPB2, ZNF451, CCNC, PRMT2, SNX2, CLTA, SNRK, LEPROT, CD9 | FHL1, YBX3, PTP4A3, TRIP10, EXOC7, HCFC1R1, ICOSLG, PLEKHF1, BHLHE40, FCGR3B, PPP1R3B, CXCR4, TLR8, TNFAIP6, FCER1G, MS4A7, VIM, VEGFA, TYROBP, ODF3B, MS4A6A, CSTA, MNDA, FCGR2C, ZEB2, CYBB, PYGL, PTPRE, LYZ, IFNGR1, PLEK, RGS1, MARCKS, FN1, SRGN, PECAM1, ADORA3, CLEC7A, GNB4, MPEG1, TNFAIP3, CXCL16, CSF1R, TAGAP, 1-Mar, DOCK4, MS4A4A, ICAM1, CLEC4A, SPARC, TUBB6, FCGR2A, FAM198B, HCK, IGSF6, AXL, FCGR1CP, DUSP4, FCGR1B, TLE3, CENPU, CSF2RA, GJA1, LILRB2, KLF4, CD14, TLR2, LILRA2, CD1D, BCL6, FAM49A, LYN, ADM, SOD2, P2RY13, SIRPA, CHST15, RAB31, TGFBI, TM6SF1, YBX3, TLR4, VCAN, KCNJ2, LILRB1, ADGRE2, LDLRAD3, VWF, P2RY1, CD33, CD93, IFI30, ADGRL4, FGR, CD300LF, CP, CD163, SPI1, SLC15A3, PTAFR, AOAH, BCL2A1, SCARF1, PELI1, NBN, GUCY1A3, ITGAX, CALCRL, LOC101928615, CSF3R, GZMB, IL13RA1, CD36, NCF2, CALD1, PTP4A1, CCL4, RNASE2, PRSS23, B3GNT5, ADAM9, PLA2G7, STAB1, QKI, RAB32, SKAP2, PTGS1, COL3A1, SIPA1L2, FPR1, CEBPB, TIMP2, CLEC12A, PLXDC2, S100A9, MAP3K8, TMEM144, FOSL2, ABCA1, KIF20B, NAMPT, COL8A1, CCL3, GAS7, GNA15, SERPINB8, BACH1, JUN, DUSP1, LILRB3, CST3, CDKN1A, CNRIP1, SLC2A3, SLC2A3, CCRL2, FSTL1, DOCK5, EDN1, LILRA1, OLR1, KCNE3, BASP1, GLUL, ETS2, VNN2, PPP1R15A, PLAUR, HSPA6, RHOJ, SYTL3, RNF144B, PDE4B, CFP, RHOB, HMCN1, FGFBP2, ARHGEF7, TM4SF1, RBP7, SIRPB2, SOCS3, FPR2, MAFB, NLRP3, THBD, COPA, SAMSN1, CEBPD, MCTP1, C15orf48, C5AR1, AIF1, PMAIP1, IL1B, MSRB3, CPE, CCL20, |

**Table S6. List of shared key genes (sKGs) from PPI network based on different topological measures**

| SN | **List of shared key genes (sKGs) from PPI network based on different topological measures** | | | | | | | |
| --- | --- | --- | --- | --- | --- | --- | --- | --- |
| 1 | **SKGs** | **Betweenness** | **BottleNeck** | **Closeness** | **Degree** | **MNC** | **Radiality** | **Stress** |
| 2 | IL1B | 12537.89 | 32 | 201.80 | 105 | 105 | 10.91 | 128538 |
| 3 | FN1 | 8263.43 | 9 | 180.77 | 69 | 68 | 10.75 | 83548 |
| 4 | JUN | 7538.00 | 12 | 175.02 | 60 | 57 | 10.68 | 65552 |
| 5 | VIM | 2677.76 | 18 | 108.05 | 20 | 32 | 8.10 | 14282 |
| 6 | TLR2 | 5277.12 | 19 | 188.63 | 85 | 85 | 10.79 | 65370 |
| 7 | TLR4 | 4465.85 | 21 | 189.27 | 86 | 86 | 10.79 | 63330 |
| 8 | CXCL8 | 4362.82 | 10 | 109.25 | 78 | 77 | 10.75 | 57026 |
| 9 | CDC42 | 1136.15 | 42 | 123.31 | 37 | 27 | 8.36 | 53826 |
| 10 | SCARB1 | 3139.38 | 24 | 118.40 | 29 | 29 | 8.30 | 23654 |
| 11 | GOT2 | 4351.82 | 12 | 113.30 | 23 | 18 | 8.34 | 16694 |

| **Table S7. The significant prognostic value of CpG in sKGs** | | | | | |
| --- | --- | --- | --- | --- | --- |
| **sKGs** | **Gene Group** | **CpG Island** | **CPG Name** | **HR** | ***P*-values** |
| TLR2 | 5'UTR | S_Shore | cg19037167 | 0.482 | 0.001697826 |
| JUN | TSS1500 | S_Shore | cg20787340 | 0.499 | 0.000716584 |
| JUN | TSS1500 | Island | cg24425829 | 0.531 | 0.001830338 |
| JUN | TSS1500 | Island | cg15995771 | 0.466 | 0.000142815 |
| IL1B | TSS200 | Open_Sea | cg07935264 | 0.513 | 0.000679216 |
| IL1B | 3'UTR | Open_Sea | cg14117934 | 0.509 | 0.000639764 |
| IL1B | 1stExon;5'UTR | Open_Sea | cg20157753 | 0.447 | 0.001210206 |
| FN1 | Body | Island | cg10692870 | 2.352 | 0.001375305 |
| FN1 | 5'UTR;1stExon | Island | cg14190674 | 0.416 | 0.000174548 |
| FN1 | TSS1500 | S_Shore | cg15127661 | 0.446 | 0.00061603 |
| FN1 | Body | Open_Sea | cg25099021 | 0.464 | 0.000307868 |
| FN1 | Body | Open_Sea | cg26581448 | 0.504 | 0.001313062 |
| FN1 | 5'UTR;1stExon | Island | cg26910092 | 0.524 | 0.001190977 |
| VIM | 5'UTR | Island | cg01154046 | 0.424 | 0.001393296 |
| VIM | Body | Island | cg12874092 | 0.326 | 0.000175551 |
| VIM | Body | Open_Sea | cg19170009 | 2.412 | 0.001732825 |
| VIM | TSS200 | N_Shore | cg20198108 | 2.071 | 0.00036068 |
| VIM | TSS1500 | N_Shore | cg23821329 | 2.206 | 0.000382225 |
| VIM | 5'UTR | Island | cg26983469 | 2.864 | 0.000981665 |
| CDC42 | 3'UTR | Open_Sea | cg07135664 | 2.396 | 0.001831628 |
| CDC42 | Body | Open_Sea | cg23019935 | 0.364 | 0.000456682 |
| SCARB1 | Body | S_Shore | cg05620762 | 0.465 | 0.000189925 |
| SCARB1 | Body | Open_Sea | cg10911287 | 0.476 | 0.00031439 |
| SCARB1 | TSS200 | Island | cg15283062 | 0.467 | 0.000674326 |
| SCARB1 | Body | Open_Sea | cg22775642 | 3.584 | 0.000128578 |
| SCARB1 | Body | Open_Sea | cg23460943 | 3.471 | 0.000361106 |
| GOT2 | Body | Open_Sea | cg04471375 | 0.469 | 0.000179944 |
| GOT2 | Body | Island | cg13626907 | 0.39 | 0.000800631 |

**Table S8:** **Docking scores (binding affinities, kcal/mol) between the proposed receptors and top ordered 30 candidate drugs (out of 148)**

| **Drug/Protein** | **GOT2** | **GATA2** | **CDC42** | **TLR2** | **TLR4** | **IL1B** | **FOXL1** | **FN1** | **FOXC1** | **NR2F1** | **CXCL8** | **VIM** | **YY1** | **SCARB1** | **JUN** |
| --- | --- | --- | --- | --- | --- | --- | --- | --- | --- | --- | --- | --- | --- | --- | --- |
| Digoxin | -9.8 | -8.7 | -8.8 | -8.7 | -9 | -8.3 | -8.5 | -9.6 | -7.9 | -8.6 | -7.7 | -7.5 | -7.6 | -7.4 | -7.1 |
| Imatinib | -9.4 | -7.8 | -7.5 | -8.1 | -7.2 | -7.4 | -7.6 | -7.7 | -7.9 | -7.7 | -7.8 | -7.1 | -7.8 | -7.2 | -7.4 |
| Dovitinib | -9.8 | -7.3 | -7.4 | -7.9 | -7.4 | -7.1 | -7.7 | -9.6 | -7.1 | -7.1 | -7.6 | -7.4 | -7.6 | -7.4 | -5.6 |
| Glimepiride | -7.8 | -7.8 | -7.9 | -7.7 | -7.1 | -7.3 | -7.7 | -7.5 | -7.5 | -7.4 | -7.9 | -7.6 | -6.5 | -6.3 | -6.1 |
| Sorafenib | -7.5 | -7.8 | -7.7 | -7.9 | -8 | -7.8 | -7.9 | -7.9 | -7.4 | -7.8 | -6.7 | -7.2 | -6.7 | -6.5 | -5.6 |
| BetaEstradiol | -7.7 | -8.4 | -7.3 | -7.4 | -7.9 | -7.4 | -7.2 | -7.7 | -7.6 | -7.3 | -7.8 | -6.6 | -7 | -7.1 | -6 |
| Vemurafenib | -9 | -7.2 | -7.5 | -7.4 | -7.7 | -7.1 | -7.5 | -7.1 | -7.6 | -7.1 | -6.8 | -6.5 | -6.5 | -6.5 | -5.8 |
| Etoposide | -8.4 | -7.8 | -7.6 | -7.4 | -7.1 | -7.1 | -7 | -7.6 | -6.3 | -6.4 | -6.3 | -7.1 | -6.5 | -7.3 | -5.7 |
| Vatalanib | -9 | -7.3 | -7.1 | -7.2 | -7.7 | -7.8 | -6.7 | -7.1 | -6.6 | -6.6 | -7.5 | -7.2 | -6.5 | -7.2 | -5.6 |
| Glibenclamide | -7.8 | -7.1 | -7.6 | -7.7 | -7.2 | -7.9 | -6.9 | -7 | -7.7 | -6.9 | -6.6 | -7 | -6.6 | -6.9 | -6 |
| Neochamaejasmina | -8.1 | -7.7 | -7.2 | -7.1 | -7.6 | -7.4 | -7.6 | -7 | -6.1 | -6.6 | -6.3 | -6.1 | -6.2 | -5.9 | -5.3 |
| Tacrolimus | -7.1 | -7.4 | -7.3 | -7.7 | -7.3 | -6.9 | -7 | -7 | -6.1 | -7.1 | -6.4 | -6.4 | -6.3 | -6.5 | -5.7 |
| Pexidartinib | -7.1 | -7.8 | -7.1 | -7.6 | -7.3 | -7 | -6.9 | -5.6 | -7.2 | -7 | -6.6 | -6.4 | -6.6 | -6.7 | -5.4 |
| Daunorubicin | -8 | -7.1 | -7.9 | -7.2 | -7.9 | -7 | -7.4 | -7 | -6.3 | -6.5 | -6.4 | -5.9 | -6.9 | -5.9 | -5 |
| Saquinavir | -7.8 | -8.2 | -7.3 | -7.4 | -8.4 | -7 | -7.5 | -5.8 | -6.8 | -6.1 | -7.2 | -6.2 | -4.5 | -7.1 | -5.1 |
| Maraviroc | -7.8 | -8.3 | -7.8 | -7.2 | -6.5 | -7 | -6.9 | -6.6 | -7.1 | -6.2 | -6.3 | -7.4 | -5.6 | -6.5 | -5.4 |
| TT301 | -7.3 | -7.6 | -7.8 | -7.9 | -6.7 | -6.7 | -6.6 | -6.9 | -6.6 | -6.4 | -6.1 | -7 | -6.7 | -6.5 | -5.6 |
| Buflomedil | -8.2 | -7.9 | -7.5 | -7.9 | -6.9 | -7 | -7.1 | -8.3 | -6.5 | -6.5 | -6.2 | -6 | -6.3 | -5.6 | -5.4 |
| Epirubicin | -8.2 | -7.1 | -7.7 | -7.5 | -6.7 | -6.8 | -7.3 | -7 | -7 | -7 | -7 | -7 | -7 | -7 | -7 |
| Doxorubicin | -7.5 | -7.2 | -7.9 | -7.1 | -6.8 | -6.9 | -7.3 | -7.3 | -6.4 | -6.4 | -6.1 | -5.7 | -6.5 | -6.4 | -5 |
| Vicriviroc | -7.3 | -8.2 | -7.6 | -6.4 | -6.7 | -6.3 | -6.9 | -6.5 | -6.7 | -6.4 | -6.4 | -6.3 | -6.2 | -5.9 | -5 |
| Sitagliptin | -7.5 | -7.1 | -7.1 | -6.8 | -6.9 | -6.8 | -6.7 | -6.9 | -6.6 | -6.5 | -6.1 | -6.3 | -6.4 | -5.9 | -5.3 |
| CDC801 | -7.4 | -7.5 | -7.1 | -6.3 | -6.5 | -6.5 | -6.6 | -7.2 | -6.3 | -6.3 | -6.1 | -6.8 | -6.1 | -6.2 | -4.7 |
| Nelfinavir | -7.3 | -8 | -8.1 | -6.3 | -6.6 | -6.8 | -5.8 | -5.9 | -6.2 | -6 | -6.1 | -6.4 | -6.2 | -6.6 | -5.7 |
| Sildenafil | -7.4 | -7.5 | -7.1 | -6.2 | -6.3 | -6.5 | -6.6 | -7.1 | -6.4 | -6.2 | -6.5 | -6.4 | -6.3 | -6.1 | -5.4 |
| BetaEstradiol | -7.9 | -7.7 | -7.4 | -6.5 | -6.4 | -6.7 | -6.5 | -6.5 | -6.2 | -6.2 | -7.3 | -6 | -6.4 | -6.1 | -5.1 |
| Sergeolide | -7.4 | -7.8 | -7 | -6.8 | -7.1 | -6.6 | -6.3 | -6.9 | -6.3 | -6.5 | -6.1 | -6.1 | -6.1 | -5.9 | -5 |
| Flavoxate | -7.5 | -7.3 | -7.1 | -6.4 | -5.8 | -6 | -6.8 | -7.4 | -6.8 | -6 | -6.5 | -5.7 | -6.3 | -6.7 | -5.5 |
| Mebendazole | -7.7 | -7.1 | -7.7 | -6.5 | -6.4 | -6.2 | -6.4 | -6.9 | -6.7 | -6.3 | -6.5 | -6.3 | -6 | -5.7 | -5.2 |
| Candesartan | -7.5 | -7.8 | -6.7 | -6.1 | -6.4 | -6.6 | -6.7 | -6.7 | -6.7 | -6.4 | -6.2 | -6.5 | -6.9 | -6.4 | -5.8 |

**References**

1. Manjusha, S., Amit, M. & Ronak, S. Association of pharmaceutical teachers of India a study on prescribing pattern and potential drug-drug interactions in type 2 diabetes mellitus inpatients. *Indian J. Pharm. Pract.* **7**, 7–12 (2014).

2. Triplitt, C. Drug interactions of medications commonly used in diabetes. *Diabetes Spectr.* **19**, 202–211 (2006).

3. Sarayani, A., Rashidian, A. & Gholami, K. Low utilisation of diabetes medicines in Iran, despite their affordability (2000-2012): A time-series and benchmarking study. *BMJ Open* **4**, 1–9 (2014).

4. Ramanathan, K., Karthick, H. & Arun, N. Structure Based Drug Designing for Diabetes Mellitus. *J. Proteomics Bioinforma.* **3**, 310–313 (2010).

5. Ambasta, R. K., Kohli, H. & Kumar, P. Multiple therapeutic effect of endothelial progenitor cell regulated by drugs in diabetes and diabetes related disorder. *J. Transl. Med.* **15**, 1–17 (2017).

6. Freshour, S. L. *et al.* Integration of the Drug-Gene Interaction Database (DGIdb 4.0) with open crowdsource efforts. *Nucleic Acids Res.* **49**, D1144–D1151 (2021).

7. Moiyadi, A. V. & Sridhar, E. δ-Aminolevulinic acid-induced fluorescence unmasks biological intratumoral heterogeneity within histologically homogeneous areas of malignant gliomas. *Acta Neurochir. (Wien).* **157**, 617–619 (2015).

8. Bielecka, Z., Czarnecka, A., Solarek, W., Kornakiewicz, A. & Szczylik, C. Mechanisms of Acquired Resistance to Tyrosine Kinase Inhibitors in Clear - Cell Renal Cell Carcinoma (ccRCC). *Curr. Signal Transduct. Ther.* **8**, 219–228 (2014).

9. Baishya, B., Satpathy, A., Nayak, R. & Mohanty, R. As a library , NLM provides access to scientific literature . Inclusion in an NLM database does not imply endorsement of , or agreement with , the contents by NLM or the National Institutes of Health . Learn more : PMC Disclaimer | PMC Copyright Notice. **23**, 163–167 (2019).

10. Atkins, M. B. & Tannir, N. M. Current and emerging therapies for first-line treatment of metastatic clear cell renal cell carcinoma. *Cancer Treat. Rev.* **70**, 127–137 (2018).

11. Kroeger, N. *et al.* Metastatic non-clear cell renal cell carcinoma treated with targeted therapy agents: Characterization of survival outcome and application of the International mRCC Database Consortium criteria. *Cancer* **119**, 2999–3006 (2013).
